# Localisation of digital health tools used by displaced populations in low and middle-income settings: a scoping review and critical analysis of the Participation Revolution

**DOI:** 10.1186/s13031-023-00518-9

**Published:** 2023-04-15

**Authors:** Jennifer Benson, Tilman Brand, Lara Christianson, Meret Lakeberg

**Affiliations:** 1 Faculty of Human and Health Sciences, Public Health, The University of Bremen, Bremen, Germany; 2 Department Prevention and Evaluation, Leibniz Institute for Prevention Research and Epidemiology – BIPS, Bremen, Germany; 3 Leibniz Science Campus Digital Public Health, Bremen, Germany

**Keywords:** Humanitarian, Localisation, Participation Digital health, Displaced populations, Digital divide, Health inequities, Low-and-middle-income countries

## Abstract

**Background:**

Forced displacement is a crucial determinant of poor health. With 31 people displaced every minute worldwide, this is an important global issue. Addressing this, the Participation Revolution workstream from the World Humanitarian Summit’s Localisation commitments has gained traction in attempting to improve the effectiveness of humanitarian aid. Simultaneously, digital health initiatives have become increasingly ubiquitous tools in crises to deliver humanitarian assistance and address health burdens.

**Objective:**

This scoping review explores how the localisation agenda’s commitment to participation has been adopted within digital health interventions used by displaced people in low-and-middle-income countries.

**Methods:**

This review adopted the Arksey and O’Malley approach and searched five academic databases and three online literature repositories with a *Population*, *Concept* and *Context* inclusion criteria. Data were synthesised and analysed through a critical power lens from the perspective of displaced people in low-and-middle-income-countries.

**Results:**

27 papers demonstrated that a heterogeneous group of health issues were addressed through various digital health initiatives, principally through the use of mobile phones. The focus of the literature lay largely within technical connectivity and feasibility assessments, leaving a gap in understanding potential health implications. The varied conceptualisation of the localisation phenomenon has implications for the future of participatory humanitarian action: Authorship of reviewed literature primarily descended from high-income countries exposing global power dynamics leading the narrative. However, power was not a central theme in the literature: Whilst authors acknowledged the benefit of local involvement, participatory activities were largely limited to informing content adaptations and functional modifications within pre-determined projects and objectives.

**Conclusion:**

With over 100 million people displaced globally, effective initiatives that meaningfully address health needs without perpetuating harmful inequalities are an essential contribution to the humanitarian arena. The gap in health outcomes evidence, the limited constructions of health, and the varying and nuanced digital divide factors are all indicators of unequal power in the digital health sphere. More needs to be done to address these gaps meaningfully, and more meaningful participation could be a crucial undertaking to achieve this.

*Registration* The study protocol was registered before the study (10.17605/OSF.IO/9D25R) at https://osf.io/9d25r.

**Supplementary Information:**

The online version contains supplementary material available at 10.1186/s13031-023-00518-9.

## Introduction

### Background

Today, the number of forcibly displaced people due to conflict, violence, climate change, and natural-hazard-related disasters exceeds 100 million [1, 2]. The majority of these are located within low and middle-income countries (LMIC) [3, 4] with health systems that can be characterised as insufficient or disrupted [5, 6]. Forced displacement is a critical determinant of poor health [7, 8] and is disproportionally protracted [9] with an average of 20 years [10]. The extended nature of displacement can result in long-term dependencies upon states who may be unable or unwilling to provide essential health and humanitarian services. International policy frameworks such as the 1951 Refugee Convention [11], International Human Rights Law [12], the Sustainable Development Goals (SDGs) [13, 14] and the Global Refugee Compact [15, 16] all aim to support displaced people “*through inclusive and comprehensive approaches*” [17]. However, the reality is that the global health burden has stretched finite response resources and failed to reach all those in need [18, 19].

In the search for innovation and improvement, digital tools have been transformative within the humanitarian health sphere [20–22]. Digital health refers to a broad range of information communication technology that, alongside experts or lay helpers or as self-help models, aims to help prevent and manage disease, as well as improve health and wellness [23]. Promoted as cost-effective and scalable tools [24, 25], they have the potential to respond to a variety of critical health challenges faced within humanitarian crises today [26]. However, the convergence of digital health tools into the humanitarian sphere is not without risk. Digital health tools have the potential to exacerbate existing societal vulnerabilities and inequalities through exclusion or discrimination. As the aim of humanitarian action is to save lives and reduce suffering, its delivery must be appropriate, timely and according to need. Addressing potential risks, International Humanitarian Law (IHL) [27] and the core humanitarian principles (HP) of humanity, neutrality, impartiality, and independence [28] provide guidance here to humanitarian practitioners in ensuring harm is not caused in the provision of humanitarian aid. The Sphere Standards [29] and the Core Humanitarian Standard (CHS) [30] provide practical support for applying IHL and HP in humanitarian action.

The introduction of digital innovations in humanitarian space, primarily a product of the Western World, is representative of a prevailing dominance within contemporary humanitarian action. Humanitarian crises mainly occur within LMIC, yet the leading response system is governed by an international set of actors and practices. This western ascendency continues despite the recognition that local actors offer relevant skills, experience, and expertise in crises [31–33]. The Sphere Standards outline active community participation as a key pillar within the Humanitarian Charter and minimum operational standards for all sectors of contemporary humanitarian action [29]. The CHS outlines that humanitarian response should be informed by participation and feedback [benchmark 4] [30]. These standards aim to centre crisis-affected people in response decision-making and rebalance uneven power dynamics in the delivery of humanitarian action.

Despite these well-accepted humanitarian standards, local responders remain marginalised by international humanitarian efforts [34–36], resulting in uneven humanitarian assistance. Paradoxically, the paternalistic humanitarian system perpetuates the same inequalities and injustices it seeks to address. This causes a *“crisis of legitimacy”* [37] for the “*broken*” international system [38], often across colonial fault lines. This paradox was discussed at the World Humanitarian Summit (WHS) in 2016 [39, 40] with calls for systemic reform [41]. From these discussions, Localisation was a major reform tenet. The *Localisation Agenda* pivoted around ten commitments aiming to collectively shift the dominant paradigm power within humanitarian responses to be “*as local as possible, as international as necessary*” [42]. One workstream to achieve localisation is the *Participation Revolution* [43]. This was outlined as a mechanism to shift power to crisis-affected people by involving them in the decision-making that affects their lives. Localisation as a set of collective commitments was promoted to recalibrate and ameliorate humanitarian action as an act of empowerment [44–48].

Participatory action is not new. There is much scholarly literature on the benefits of participation within humanitarian and health care interventions and research, spanning several decades. With a focus on digital health specifically, O’Connor, Hanlon and O’Donnell et al.’s systematic review [49] explores barriers to participation within digital health projects. They found motivation, personal values, the engagement approach, and the quality of the intervention to be key participatory obstacles and recommend more attention towards engagement strategies and personalised digital health tools. Through participation, the adaptation of health interventions to address specific groups was considered highly important in overcoming barriers in mental health. Gearing et al.’s systematic review [50] agreed with these findings and concluded that one of the main barriers was contextual unacceptability of interventions.

A recent study ‘*Nothing about us without us?’* [51] highlighted the important user involvement roles within mental health interventions in humanitarian crises. This paper concluded that current participation practices were considered “*predominantly tokenistic*” [51] and transactional, with a lack of guidance on how to carry out meaningful and ethical user involvement within interventions. However, Greenhalgh et al.’s systematic review of participation of users in research [52] found 65 different frameworks developed to assess and guide participation. These were grouped by type according to purpose for identifying power, priorities, studies, reporting and partners [52]. For assessing engagement in health research, they outlined principles of patient-centredness, using plain language, shared responsibility for the study and its outputs, checking assumptions, ensuring meaningful community outcomes, and partner skill building through the process [52].

Arnstein’s *Ladder of Citizen Participation* [53] conceptualises each rung as a greater extent of interaction and greater power gains. The ladder is split into three main groups; the first is non-participation, which includes manipulation, therapy and informing. The second group is degrees of tokenism, which includes consultation and placation. The third group, the highest on the ladder, includes partnership, delegated control and ultimately citizen control. The CDAC Scale of Community Engagement [54] mirrors the ladder framework with its five stages of increasing interaction with communities and aims for community leadership as the gold standard. The commonality between these frameworks is they all outline that best participatory action is inclusive, deep, and multi-faceted, where powershifts can affect process outcomes.

This aligns with the increasing humanitarian awareness of power dynamics and ethical approaches to research and action. In recent years there has been a greater focus on accountability and engagement [55–57] with standards set up for protection and inclusion of specific community groups, all with the aim to better protect and provide for those affected by humanitarian crises.

Despite the ongoing empowerment and effectiveness narratives, climate related emergencies and the unequal global Covid-19 response highlight some of the pervasive structural inequalities within the international humanitarian and health systems [58]. As recognition and acknowledgement of racism, discrimination and colonisation grows in the global consciousness, following key worldwide events and movements, humanitarian and health communities are being confronted by the systemic injustices that underpin them [59]. The Localisation Agenda has parallels with anti-racism and decolonisation movements in calling for a shift in power toward those disproportionately affected by colonial consequences [60, 61].

### Rationale and objectives

If power is to be shifted then the Localisation Agenda must become more than just rhetoric: Action should be reparative and empowering and break free from traditional binds of power [62]. There are several local organisations that have emerged to progress the Localisation Agenda as part of their work: The Charter for Change (C4C) [63] is a collection of over 50 international organisations that have committed themselves to localise humanitarian aid. The Network for Empowered Aid Response (NEAR) [64] is a network movement of local and national societies that promote and advocate for equitable partnerships within humanitarian aid. The Alliance for Empowering Partnerships (A4EP) [65] supports sustainable cooperation underpinned by the Universal Declaration of Human Rights. The Global Refugee-Led Network [66] advocates for greater representation in global humanitarian aid decision-making. These organisations all demonstrate an appetite and capability for locally led crisis solutions. Despite this, global health and humanitarian policy efforts have largely continued with a top-down approach [67, 68]. This is illustrated by the seven dimensions of localisation as laid out in the NEAR Localisation Performance Measurement Framework [69], and the Global Mentoring Initiative’s Dimensions of Localisation Framework (GMI) [45]. These widely accepted frameworks outline localisation through domains of partnerships, funding, capacity, coordination, policy influence and visibility and participation. Collectively, these systemic dimensions frame localisation as a technocratic fix. Whilst this approach may be applicable to a diverse set of organisations it falls short of capturing the intersectionality of the power paradigm within the reality of humanitarian assistance.

Since the humanitarian imperative of health action is to save lives and reduce suffering in crises [70], it is a worthy endeavour to reorientate ourselves to the crisis level to contribute to scholarly critique within this domain. Focusing in on one of these dimensions, participation, due to its greatest proximity to crisis-affected people, we enquire through a power lens: How has the Participation Revolution been adopted within digital health interventions for displaced people within LMIC? This study seeks to investigate this phenomenon.

Applying a scoping review approach to map out, synthesise and critically explore existing scholarship through a power lens, this paper aims to investigate the participation of displaced people within digital health humanitarian efforts. To achieve this, we investigate (A) the characteristics of digital health tools used by displaced people in LMIC; (B) the characteristics of the interventions themselves (C) how and to what extent participatory action has been carried within the development and implementation of these projects.

## Method

A protocol was written and registered before the study (10.17605/OSF.IO/9D25R) at https://osf.io/9d25r [71].

### Inclusion and exclusion criteria

A Population, Concept and Context (PCC) framework [72] was developed as the inclusion criteria (Table 1).

**Table 1:** PCC inclusion and exclusion criteria

	Inclusion criteria	Exclusion criteria
Population:	Humans of any demographic background that have been internally or internationally displaced following a humanitarian crisis (conflict, disaster, outbreak, etc.)	Non-human samples, economic migrants, expatriates, and other migratory groups
Concept:	Digital health interventions, public health perspectives, participatory digital health methods, localisation & shifting power	Digital interventions not associated with health outcomes, digital interventions not applied (e.g., digital prototype development, not conducted), digital interventions not used directly by displaced populations (e.g., digital interventions used by clinical humanitarian/health responder
Context:	Low-and middle-income countries and contexts as defined by the World Bank [130] as the location displaced people find themselves in seeking humanitarian assistance	Studies not carried out in, or discussions not referring to low resource settings
Other:		Studies published in languages other than English that cannot be auto translated to English, full texts not accessible following extensive search efforts and literature published before 2010

The study types included were relevant peer-reviewed and non-peer-reviewed primary interventions, secondary research, and relevant discussions (books, scholarly articles, discussion and opinion pieces, grey literature, non-scientific interventions, reports, policy, and guidance documents).

The 2010 data limitation was added to exclude obsolete technologies with the aim that resulting discussions would be reflective of current practice.

### Search strategy

The search was conducted in English with no limits on five academic databases. A search syntax combining keywords and subject terms using Boolean operators was developed with the support of a professional librarian (LC). The following concepts were searched: *displaced population, digital health, localisation,* and *low, middle income* (Additional file 1: search syntax). The selected databases were CINAHL via EBSCO, Medline via PubMed, PsycINFO via OvidSP, Sociological Abstracts via ProQuest and Web of Science Classic via Clarivate Analytics. These databases were selected in collaboration with a professional librarian (LC) to provide a multi-disciplinary life and behavioural science result. In addition, three online grey literature repositories were searched using the search terms; *digital, local and health* within the International Committee of the Red Cross’s resource portal (ICRC) [73], Oxfam’s Open Library Repository [74] and the Active Learning Network for Accountability and Performance’s resource portal (ALNAP) [75], to source relevant publications. These repositories were selected by the lead author (JB) due to prior use and understanding of their relevant content according to the key words. Other repositories were also considered for inclusion however the manual nature of the search and export functions was timely, and a decision (JB & TB) was made to limit their use to three. A Cochrane LMIC filter [76] was applied to the academic databases, and a date filter from 2010 onwards (final database search date—29.09.2021 and final repository search date—09.11.2021) was applied to all searches.

### Screening and data extraction

We adopted the five-step approach as outlined by Arksey and O’Malley [77]; *(1)* identification of the research question, *(2)* identification of relevant studies which were entered into EndNote20 and then imported into Covidence. Deduplication and study selection took place through *(3)* two levels of screening by two independent reviewers (JB and ML), title and abstract screening and full-text review resulting in 27 papers for inclusion. Conflict resolution discussions were held to come to a consensus on disagreements. *(4)* Microsoft Excel data charting summarised all papers (Additional file 3: data chart) and centred around the literature characteristics, as outlined within the protocol [71]. This process was subject to a quality control review by both reviewers (JB & ML) independently at the extraction stage. *(5)* The identification and charting of reported localisation and participatory action and associated emerging themes followed iteratively (JB and ML) as an extension of the main chart to form the basis of the paper’s results section. Additional literature was drawn upon to further the team’s comprehension and knowledge of key themes and to situate the paper within a broader body of literature. This was cited throughout the paper but not charted within the literature table. The PRISMA 2020 checklist guided the scoping study’s write-up [78] (Additional file 5: PRISMA checklist).

### Data synthesis and analysis

The review took a critical stance toward analysis through the idea that reality is the product of language, social roles and forces of power [79, 80], grounded within practical contexts rather than being developed in isolation [81–83], we identify the literature as steeped in power dynamics (interpretivism).

We borrowed from Molinengo’s *Flows of Power Framework *[84] and define power as both acts and flows: *“An act of power is the capacity of an actor to intervene at a specific moment during a collaborative process, … according to their own interests”* [84] and *“A flow of power is a chain of actions, originating from one initial act of power … that contribute to the ongoing interplay between designed and emerging interaction orders” *[84]*.*

Within the literature we looked for synergies and alignments with the *Emerging Indicators of the Participation Revolution* (see Additional file 6), as mentioned within the GMI *Dimensions* of *Localisation Framework *[45] and the *Participation Key Performance Indicators* within the *NEAR Localisation Performance Measurement Framework* [69] as a process of identifying how the Participation Revolution has been enacted.

We applied Arnstein’s Ladder of Citizen Participation as a power lens [85] to interpret the extent of participatory action in the literature. We used Hilsdon et al.’s critical thinking model of description, analysis and evaluation [86, 87] to stimulate thinking, questioning and reflection to deconstruct and reconstruct meaning with this focus.

### Reflexivity

Recognising the privileged position of the research team in the interpretation of data and knowledge-making through the process of developing this paper, a reflexivity statement was drawn up. The aim of this was to provide transparency and acknowledge the power dynamics within our identity and positionality that have impacted and enabled the production of this paper, along with our interpretation of results (Additional file 4: Reflexivity statement).

## Results

### Overview

The original literature search yielded 3199 papers, 1093 from academic databases, and 2106 from grey literature sources. 142 duplicates were removed, leaving 3057 (96%) papers for screening and selection. Application of the eligibility criteria at the first screening level excluded 2965 papers, and the second excluded 92 articles (Additional file 2: PRISMA-ScR flowchart). The primary reasons for exclusion at the second screening level were; wrong intervention (n = 45), lacking a health focus, and, or the wrong population, lacking a focus on displaced population groups in LMIC (n = 11). N = 27 eligible papers remained, and all were included in the review.

### Literature and crisis characteristics

The literature provided a heterogeneous range of academic and grey literature focusing on digitalised humanitarian health. 70% of the literature were peer-reviewed academic papers (n = 19) (89–106), with the majority of these being published within the last four years (additional file 3: Data chart).

Mixed methodologies, such as, qualitative surveys, quantitative phone surveys, a cross-section study, a feasibility randomised control trial, and literature reviews were all present. N = 13 papers were general papers without interventions.

**Fig. 1:**
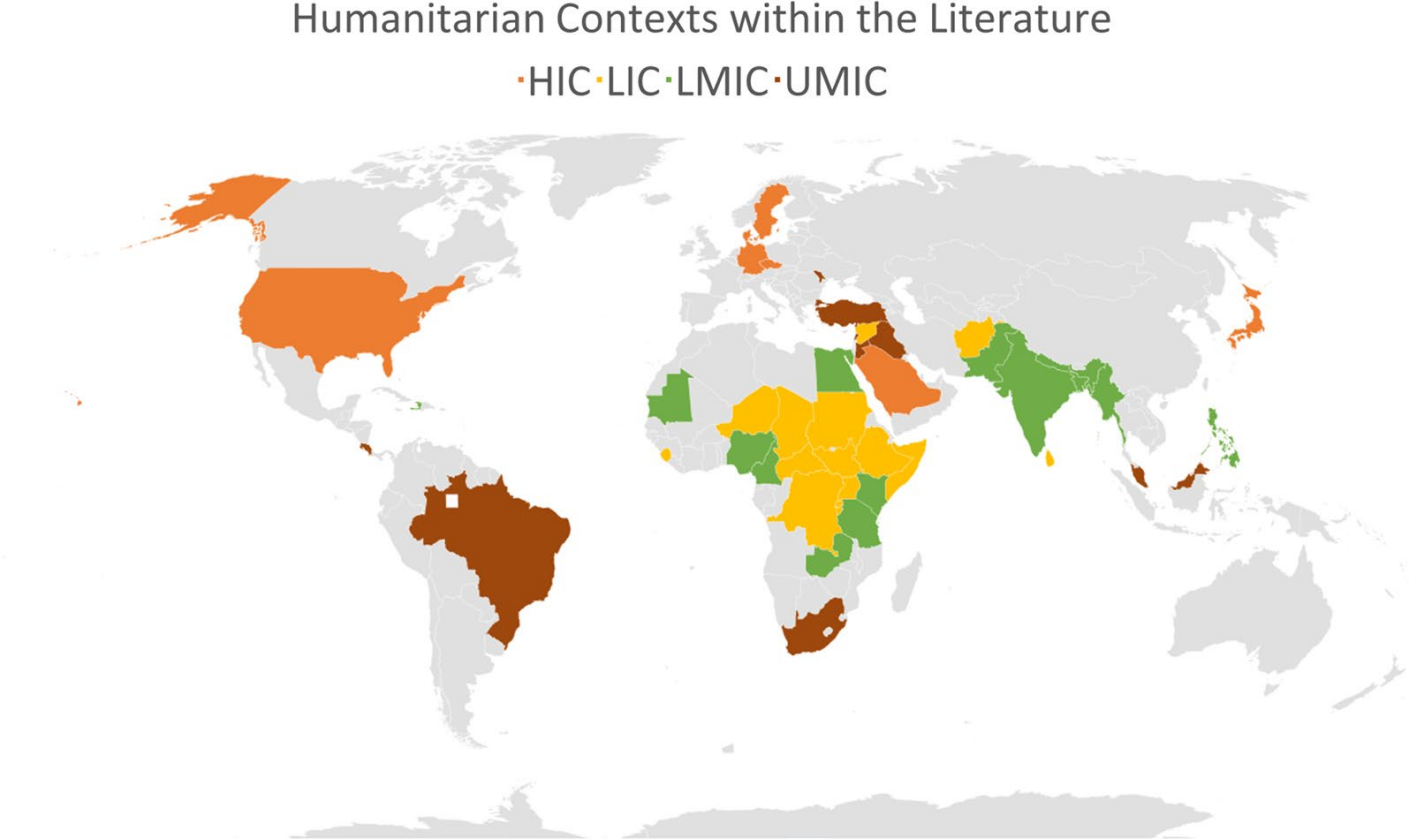
Humanitarian contexts focused on within literature, categorised by economic classification

Refugees were the primary population of interest (n = 14). This is at odds with global displacement statistics outlining a greater number of internally displaced populations (IDPs) in need of humanitarian assistance [4, 88]. There were 48 humanitarian contexts mentioned in total (Fig. 1), and several papers referred to more than one crises. According to the World Bank economic classifications n = 15 were low-income countries; n = 16 were lower-middle-income countries; n = 10 were upper-middle-income-countries (outlined in Table 2). Within the literature, certain high-income contexts were included in combination with relevant LMIC contexts (n = 7), with results that were not always disaggregated by country.

**Table 2:** Literature characteristics

Characteristic	Types	Literature reference
Literature type	**Primary study**	
	Cross-sectional	[92]
	Mixed methods	[131]
	Qualitative studies	[98, 108, 95, 90, 107–110]
	Randomised control trial	[94]
	**Secondary study**	
	Case study	[89]
	Literature review	[100, 124]
	Discussion paper	[26, 132, 99, 101, 107, 93, 105, 102, 111, 132–134]
	Other	[91]
Displacement type	Internally displaced people	[98, 89, 110]
	Refugees	[90–93, 99–103, 107–109, 102, 135]
	Both refugee and IDPs	[26, 94, 111, 134]
	Non-specific/not specified	[105, 112, 133]
Humanitarian contexts	Afghanistan	[100, 124, 112, 134]
	Bangladesh	[99, 112, 134]
	Brazil	[134]
	Burundi	[109, 134]
	Cameroon	[134]
	Central African Republic	[134, 106]
	Chad	[134]
	Chechnya	[124]
	Costa Rica	[124]
	Czech Republic	[111]
	Democratic Republic of Congo	[124, 105, 109, 134]
	Denmark	[100]
	Egypt	[100, 93]
	Ethiopia	[105, 109, 134]
	Germany	[100]
	Haiti	[89, 111, 106]
	India	[111]
	Indonesia	[106]
	Iraq	[100, 124]
	Japan	[133]
	Jordan	[90, 101, 101–103, 102, 134, 136]
	Kenya	[105, 109, 111, 112, 134]
	Lebanon	[132, 108, 100, 94, 105, 134, 137]
	Malaysia	[111]
	Mauritania	[105, 134]
	Myanmar	[99]
	Nepal	[111]
	Niger	[105, 112, 134]
	Nigeria	[109, 134]
	Pakistan	[124]
	Palestine	[98, 131, 124, 92]
	Philippines	[111, 133]
	Republic of Moldova	[111]
	Rwanda	[109, 111, 134]
	Saudi Arabia	[100]
	Sierra Leone	[133]
	Somalia	[124, 109, 112]
	South Africa	[95]
	South Sudan	[109]
	Sri Lanka	[100]
	Sudan	[100, 124, 109]
	Sweden	[100]
	Syria	[108, 100, 124, 90, 100–103, 94, 103, 110, 102]
	Tanzania	[109, 134]
	Turkey	[100, 91, 107, 109, 102, 134]
	Uganda	[109, 134]
	USA	[133]
	Zambia	[109, 134]
	General/not specified	[26, 132, 95, 124, 105, 111, 132–135]
Crisis type	Conflict	[90–94, 98–103, 93, 94, 103, 110, 102]
	Natural disaster	[89]
	Non-specific/multiple types	[26, 132, 95, 105, 109, 111, 112, 134, 135]
Targeted groups	Mental health sufferers	[98, 93–95, 98–102]
	Non-communicable disease sufferers	[96]
	Parents	[90]
	Women/girls	[92, 103]
	Non-specific groups	[26, 132, 131, 108–112, 110, 131–135]
Displacement setting	Refugee camp	[131, 99, 108, 90, 92, 109]
	Within host communities	[91, 107, 110]
	Both camp and host living	[98, 101, 134]
	Non-specific/not specified	[26, 132, 93–95, 124, 93, 94, 103, 105, 102, 111, 112, 133, 135]
	Mixed (Rural & urban)	[98]
	Non-specific/not specified	[26, 89–95, 99–103, 93, 107–112, 111, 132–135]

Reflecting major humanitarian crises within the review’s timeframe, African contexts (n = 20) were the most frequently mentioned. However, in terms of interventions, Syrian refugees in neighbouring countries (Turkey: n = 6, Lebanon: n = 7, Jordan: n = 8) were the most frequently assessed.

The majority of the literature focused upon conflict crises, with the exception of one paper [89] that focused on a natural disaster. The other papers discussed crises in general (n = 7). Displaced people and refugees were identified in camps as well as within urban and rural host populations. Further characteristics are charted in Table 2.

### Digital tools and health characteristics

The review identified a broad spectrum of digital health tools used within the humanitarian landscape, with several papers citing more than one tool. The tools were predominantly orientated towards mobile phones, with phone age and type varying from very basic mobile phones with limited functionality to more modern smartphones with a range of applications. Despite this, the most frequently cited format were smartphone applications (n = 5) [90–94] followed by short-messaging systems (SMS) (n = 3) [95–97].

Targeted or potential digital health users were parents (n = 1), women and girls (n = 2), mental health sufferers (n = 9) and NCD sufferers (n = 1). Digital health tools were set to address a range of health issues through both guided and self-help approaches. Almost half of the literature (n = 13) addressed specific health issues through a vertical approach with health promotion, preparedness, and planning, as well as responsive health management interventions. The most frequently cited health issue was mental health (n = 9) [89, 93–95, 98–102], including depression, post-traumatic and psychological stress. Maternal, reproductive, and childcare (n = 3) [90, 92, 103] followed: These interventions focused on family planning and newborn vaccinations. Communicable diseases, a common concern within humanitarian environments [104], were identified in n = 3 papers [91, 97, 105], focusing on Cholera, Covid-19 and other infectious disease outbreaks. Despite the high non-communicable disease (NCD) burden in LMIC [104], only one paper addressed NCDs with a focus on hypertension and diabetes [96] which may be reflective of the short term nature of humanitarian assistance. General health care followed this (n = 1) [106], including first aid, health promotion, and gender-based violence prevention (n = 1) [107]. There was no physical activity, nutrition interventions, or interventions targeting obesity, tobacco and drug cessation, or HIV/AIDS management that might be expected considering their respective worldwide health burdens. Within these papers, health statistics were presented or cited as a rationale for prioritising health issues, however there was no indication if consultations with targeted groups had occurred to confirm this decision-making. Further characteristics are charted in Table 3.

**Table 3:** Digital tool and health and characteristics

Characteristic	Types	Literature reference
Health issues addressed	Cholera	[105, 111]
	Covid-19	[91]
	First aid	[133]
	Gender-based violence	[107]
	General health	[133]
	Health records	[132]
	Malarial prevention/care	[111]
	Maternal health/family planning	[92, 103]
	Mental health	[98, 93–95, 98–102]
	Non-communicable diseases	[137]
	Vaccine-preventable diseases	[90]
	Unspecified	[26, 132, 108–112, 111, 112, 133, 135]
Digital tool	Mobile phone	
	SMS	[95, 107, 137]
	Application	[90–94]
	Mobile phone (Medium not specified)	[100, 109, 112]
	General	
	Telephone line	[99, 108, 89, 101, 102]
	Social Media	[98]
	Unspecified	[26, 132, 103, 105, 110]
	Mixed tools	[124, 111, 133, 135]
	Cloud-based storage systems	[132, 101, 105, 97]

### Digital health interventions

Evidence of direct health outcomes generated from digital health tools was extremely limited. Included papers tended to focus on understanding the digital landscape through baseline and pre-intervention feasibility assessments. Self-reported results found widespread infrastructure and access but intermittent connectivity (internet, electricity, network), with some areas having no coverage [92, 93, 95, 98, 100, 101, 107–110]. A high digital device ownership was found, with mobile phones being the most frequently owned devices. Digital literacy levels were limitedly explored and in some cases digital device ownership may have been conflated with digital literacy [109].

Interest, willingness and readiness to use newly introduced health tools were all self-reported as high [91–93, 95, 96, 100, 107]. User perceptions of potential health impacts through digital health tool use were assessed and reported positively. However, as one maternal care information study found, from 1042 participants, only 17% had used the digital health tool by the end of the intervention [92].

Cost was a critical factor in influencing user behaviours [91–93, 95, 96, 100] and there were quality concerns for content accuracy and sufficient user literacy levels [93, 100, 103, 107]. Social media, including Facebook, Facebook Messenger and WhatsApp were frequently used by participants but not necessarily for health information.

Findings were gendered in certain studies, with females being less likely to be digital owners than males. Additionally, older generations, those without education or low-income groups were less likely to own mobile phones [109]. Sharing behaviours broadened mobile phone usership between family members and within friend circles. However, restrictions in shared device usage or usage monitoring were reported at a low level, cited as a protection mechanism to avoid shameful online behaviours or to avoid distractions during studies [107].

### Participation within digital health tool projects

According to the information provided, there were no explicit localisation efforts within this review and it was not possible to identify Participation Revolution indicators from either the NEAR or the GMI Localisation Frameworks [69] within the literature. Localisation terminology such as empowerment language was limited but identified in n = 6 papers [91, 103, 106, 108, 111, 112].

Participation was not a central theme in the literature. Displaced people were not discussed as project partners or leaders of the digital interventions but as *participants*, *patients* and *users.* Furthermore, there was no mention of local groups involved in the conception of the interventions, their assessments, interpreting the results or preparation of the literature.

Instead of critical decision-makers and response leaders, displaced and crisis affected people were relegated to participation through answering surveys, responding to interviews and partaking in focus group discussions (FGDs) [92–96, 98, 100, 107–110]. Outside of these roles, displaced people acted as translators, interviewers [95] and site surveyors [98]. One paper [93] acknowledged that “*user experience focus is still rare”*, another stated *“no participants were involved in the development of the research questions or outcome measures or the design and conduct of this study*” [92]. Despite their limited involvement, n = 23 (85%) papers anecdotally recognised the benefit of local participation to improve intervention outcomes, citing it as a recommendation or as a lesson learned.

Results from participation activities were reported but without great attention which challenged the analysis of power within or surrounding them. Their gap in the literature is conspicuous and steeped in power inequities. Digital health tools were adapted [93] to benefit displaced people through functional and content modifications: These were motivated by user feedback on comprehension and relevance which were said to build provider credibility and trust. Participation raised the issue of user literacy levels, with one intervention team being requested to read their survey out loud to 70% of respondents [98].

Furthermore, a discussion theme focused upon construction of health and wellness concepts and how language was used, particularly in relation to cultural influences and displacement contextualisation was present. Here the importance of capturing context and cultural idioms and their gendered reality within health constructions, and handling stigmatised health issues with cultural consideration to avoid insensitive approaches [100] was outlined. These discussions included the importance of tone and accent in language [107] and not simply the language selection itself as having an impact on provider-recipient interactions. One paper said of diverse humanitarian communities *“differences across contexts are significant enough to affect population level behaviour and epidemiology”* [91]. These discussions are integral to the success of digital health but remained limited within the review as a whole.

### Authorship

**Fig. 2:**
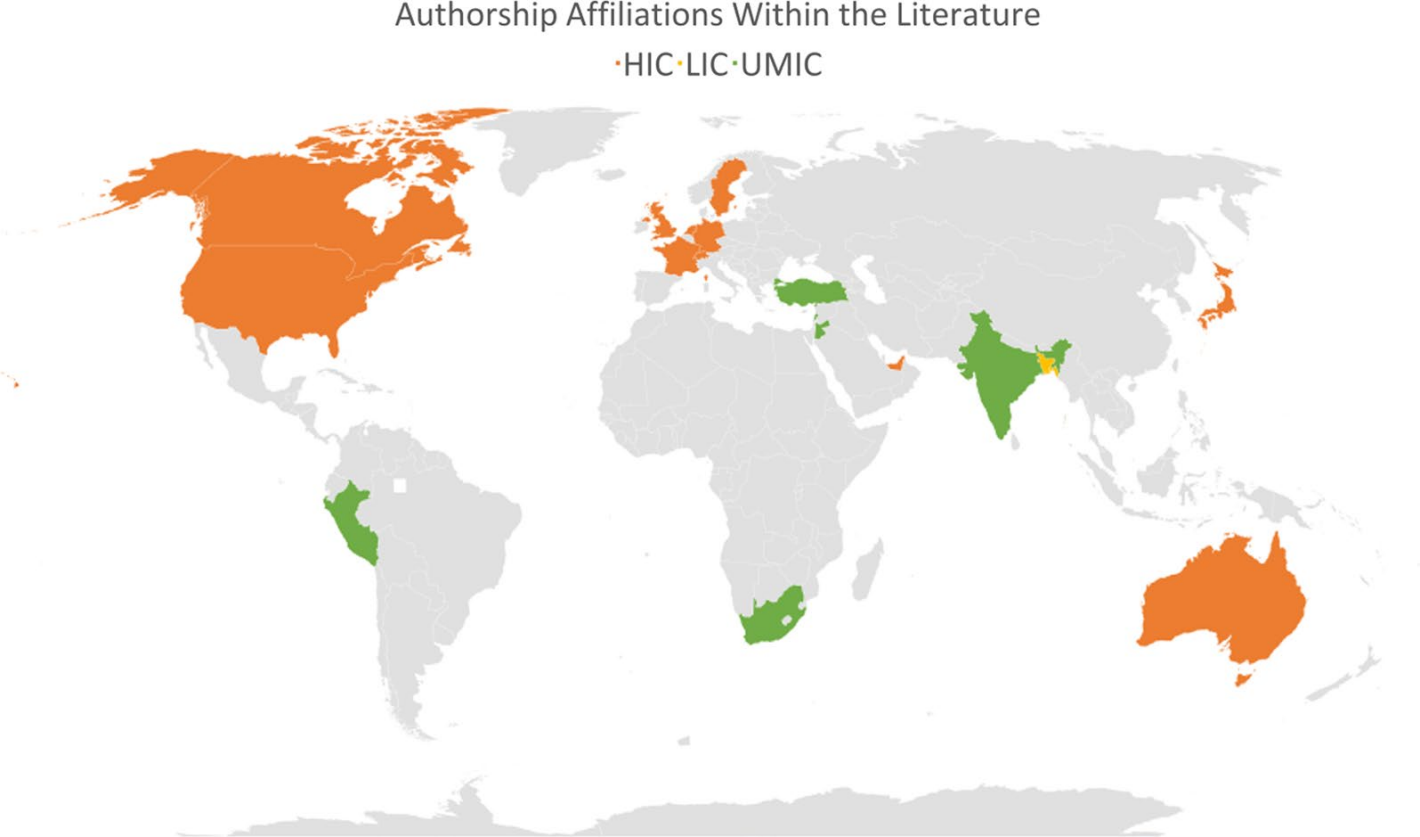
Authorship affiliations within the literature, categorised by economic classification

LMIC authorship was limited (Fig. 2). According to the World Bank economic classifications [113], only one paper had authorship affiliations from a low-income country (LIC) [Bangladesh [99]]. Six upper-middle-income authorship affiliations countries (UMIC) were present [India, Jordan, Lebanon, Peru, South Africa, and Turkey]. N = 15 authorship affiliations from HIC countries were present, including organisations or institutions based within HIC (4 of these did not explicitly state the authorship country and were written by international organisations; two from UNHCR, one from UN OCHA and one from Samuel Hall). While it is possible that displaced diaspora from LMIC contributed to the literature’s HIC authorship, there was no reflexivity in this respect.

## Discussion

### Principle findings

The objective of this study has been to explore digital health tools used in humanitarian environments by displaced people, their purpose and how they have localised their work through the participation tenet. To our knowledge, this is the first review of its kind focusing on this topic. It contributes knowledge in the arena of localisation and participation within digitalised health interventions for displaced people in LMIC. Main findings include a varied range of digital tools addressing a variety of health issues. Largely, these tools were developed with limited engagement of displaced people to achieve pre-determined objectives and without consideration of the Participation Revolution indicators. Intervention assessments in this digital realm appears to be in its infancy and not yet at the stage of evidencing health outcomes. Greater engagement with digital tool users at all project stages should be a key focus for digital health projects delivering humanitarian and health action.

### Digitalised humanitarianism

The humanitarian landscape is becoming increasingly digitalised. Despite being a western construct, *“the development of mobile phone networks in LMIC has even superseded infrastructure development of roads, electricity and traditional internet deployment”* [100]. The heterogeny of health issues and digital tools found within this review reflects other relevant reviews and echoes the fast-paced development, cost and ubiquity of innovative mobile technologies [95, 114, 115]. With two exceptions [91, 92] mobile phones were not found to be a current source of health information, however their ubiquity for many displaced people could signal positive implications for future digital health tools in their use as a health source.

### Digital divide

Despite their ubiquity, it remains unclear if digital health tools actually extend the reach of humanitarian health services. Interventions occurred around established health centres and camp catchment areas, with participants being recruited from within. Whilst the remote nature of digital health tools has the potential to benefit hard-to-reach groups such as the physically impaired or excluded groups without existing access, there was a gap in the literature here. This left unanswered questions on whether these participants, with ready access to health centres and high mobile phone ownership, are in fact the most vulnerable.

The gendered and nuanced reality of the *digital divide* [116] highlights the existing inequalities in accessing health care: Low mobile phone ownership among certain groups of women, low literacy skills among the poorly educated, prohibitively expensive data costs for the unemployed or low-income groups, and poor connectivity infrastructure for rurally located communities all sets to exclude these groups from the benefits digital health tools may offer. The inequitable and intermittent digital landscape, alongside varying literacy levels, is a critical threat to the success of digital health tools within LMIC contexts. This divide was reiterated by the UN Secretary-General in 2020, describing it as *“a matter of life or death”* [117] and this review has not provided evidence transcending these challenges (Table 4).

**Table 4:** Literature authorship origin, categorised by economic classification

Country of authorship	Literature reference
**Low-income countries**	
Bangladesh	[99]
**Upper-middle-income-countries**	
India	[99]
Jordan	[90, 103, 138]
Lebanon	[132, 108, 124, 94, 103, 96]
Peru	[102]
Turkey	[91, 107]
South Africa	[95]
**High-income-countries**	
Australia	[108, 107]
Canada	[96]
France	[107]
Germany	[93, 94]
Japan	[92]
Sweden	[108, 90]
Switzerland	[93, 94]
The Netherlands	[93, 94]
UK	[108, 124, 110–112, 112]
USA	[132, 100, 89, 101, 100–102, 105, 102, 96]
United Arab Emirates	[96]
**Unspecified authorship location (international organisations)**	
IFRC	[133]
Samuel Hall	[109]
OCHA	[26]
UNHCR	[134, 135]

### Intervention outputs

The majority of intervention literature focused on the feasibility of digital health tools, rather than testing them as a disease prevention or health promotion tool. Mapping the digital landscape and understanding connectivity barriers are acknowledged as fundamental pillars for the success of digital health. However, there is a critical knowledge gap of digital health tools improving health outcomes. It is recognised that research in humanitarian contexts is challenging, but as improving health outcomes is the digital health raison d'être, this should be a primary driver for developing digital health tools. The current focus of the digital health movement indicates its formative stage and has some way to go before digital health outcomes can be evidenced.

Whilst health outcomes within digital health are under-researched, the literature offers a range of consistently positive results indicating that displaced populations are both interested and willing to use digital tools. However, much of the research findings hinge on short-term, self-reported, unverified data with varying sample sizes and follow-up efforts. Potential response and social desirability biases were highlighted [96, 107] but not addressed with the literature and risk undermining these formative assessments. As one study demonstrated, the presence and knowledge of digital health tools are not enough to motivate their uptake [92]. As a relatively novel sphere of humanitarian health action, further research verifying these self-reports and addressing biases is an essential step to strengthening research in this field.

### Authorship reflexivity

Though localisation debates have been criticised for polarising an international-local binary that fails to recognise the hybridity and diversity of contemporary humanitarian and health action [118–120], identifying dominant voices within the literature can reveal prevailing positionality and associated power structures. Within the literature, several papers included direct quotes from displaced people, extending their voices to the reader. However, the dominance of HIC authorship within this review exposes the prevalence of knowledge-makers forming narratives through their positionality in interpreting study results with limited reflexivity.

It is possible that LMIC displaced diaspora may have been included alongside HIC researchers or implementation teams, however, there is little explicit evidence of this. This lack of attention towards positionality is problematic in that the international narrative here, whilst dominant, may not be genuinely representative of displaced people within LMIC. Addressing the positionality of this paper, the authorship team are from HIC and have not been personally affected by humanitarian crises. We recognise our prevailing perspective on this topic (Additional file 4: reflexivity statement) as a limitation in getting to the heart of localisation from the perspective of displaced people in LMIC. Nevertheless, we use our power to direct attention to this critical point and to encourage LMIC voices on this topic.

### Participation in digital health tool projects

There is an almost-universal acceptance amongst the humanitarian community that localisation and participation are positive steps towards improving response efficiency. Anderson et al.’s book, *Time to listen: hearing people on the receiving end of international aid* [121], reflects this with aspirations for a humanitarian system that moves away from crisis-affected people as passive aid recipients and towards a broader collaborative experience. This was echoed within the literature’s narrative as advantageous for developing relevant, credible digital health tools. However, the reality of this activity within the literature was either not present or contained. Characterised by Arnstein as *“an empty ritual”*, [53], local groups and individuals tended to be used as instruments of the research teams for the purpose of extrapolating information for pre-defined questions, seeking to explore pre-determined objectives including informing adaptations and functional modifications rather than leading actors, co-creators or partners within their response. The Participation Revolution, according to NEAR and GMI indicators has yet to emerge.

Intervention literature tended to be short-term, characteristic of rapid humanitarian responses with limited discussion of sustainable, integrated solutions. As a result, participatory action appeared as the intermittent involvement of small participant groups conducted by research teams. Little is written regarding the representability of these groups with the proposed digital health users or the wider crisis affected population, or the inclusion and exclusion criteria for their selection, how they were selected or whether they received remittance for their participation. Others reflected this with similar experiences of anecdotal or minimal involvement of displaced people, primarily centred around providing local knowledge and language skills to inform already pre-determined interventions [122, 123]. Bowsher’s systemic review [124] outlined that displaced people are usually excluded from digital innovation development which fails to maximise on the potential of digital tools. Scholarly reflection in this area would be helpful and could provide evidence of representability as a quality indicator within intervention studies.

Applying Arnstein’s Ladder of Citizen Participation [53], we can explore the extent of participatory activity. Within this review participatory action ranges from the very bottom of the ladder, with one paper outlined zero participatory efforts, and moving up to the second group, the ‘*degrees of tokenism’* realm. This includes *information provision*, *placation*, and *consultation *[53]. Participatory action here can be characterised as singular flows of communication offering little feedback or influencing power. Consultations were measured as “*statistical abstractions*” [53] in number of surveys completed or number of downloads. As Arnstein outlines at this level, targeted groups participated but did not necessarily benefit from their involvement, they could not influence digital health action past the extent offered by those holding the power [53].

Whilst none of the literature claims to enact localisation action, the absence of meaningful local contributions to digital health development indicates ongoing asymmetrical power relations within the humanitarian and health systems remain as prevalent as ever. Reflecting upon this gap highlights the current system's inequities. Without meaningful, representative, on-going participation from displaced people directly at all stages of interventions, the humanitarian community perpetuates a *them* and *us* narrative that risks characterising *them* as passive victims and beneficiaries without agency, capacity, or self-determination. This ‘othering’ may marginalise or harm them [125] and have exclusionary implications for the future of humanitarian and health action.

It is recognised that research and interventions in humanitarian environments can be challenging and prohibit regular and deep engagement with displaced people. Participatory action takes time, money, and energy to undertake which is often in short supply within humanitarian emergencies. However, findings from a 2020 Professionals in Humanitarian Assistance and Protection workshop [126] with 541 practitioners and experts exploring inclusive participatory humanitarian action illuminated that the leading barrier was donor pressures and donor inflexibility alongside time constraints (43%) and the greatest facilitator was staff commitment (57.7%) and staff capacity (41.6%) [126]. These indicators could become key advocacy agendas for future participation champions.

### The problematic conceptions of localisation

The general and technocratic conceptualisation of localisation enables varied systemic manifestations to evolve within the humanitarian sphere. Rather than placing displaced people at the centre of the action, the localisation dimensions risk becoming organisational tick-boxes that can be achieved through superficial exercises in their name. This facilitates certain self-serving appropriations of participatory action that suits domineering health and humanitarian actors, their modus operandi [102] and maintains the status quo.

The lack of meaningful engagement with displaced populations in digital health projects opens them up to the risk of instrumentalising techno-colonialism [127]. Madianou defines techno-colonialism as the embedded power disparities between humanitarian organisations and crisis-affected people encapsulated within digital tools [127]. Within the participation sphere, the dominance of HIC narrative found within the literature of this review appears to be primarily underpinned by western values from prominent nations and LMIC voices are rarely heard. The limited focus on selected health models and conceptualisations of health and wellness within these tools highlight tension with the principles of independence and impartiality that humanitarianism supposedly enshrines.

Whilst the Western World of health brings with it some examples of globally renowned practices and policies, these are not always appropriate or appreciated outside their context. Adopting western health models within digital tools for LMIC is therefore potentially problematic. Firstly, this reflects colonial practices of rolling out western influence at the expense of local people. Secondly, western ideals do not always translate well with differing contexts and do not always align with cultural beliefs underpinning illness and disease health constructs or routes of health care. Cultural and gender influences were addressed in several papers, but this was not widely representative of the literature’s consciousness. Further and more meaningful involvement with local groups could be the key to re-conceptualising the models of health adopted within digital tools. Without this, digital health tools may reproduce inappropriate health constructions rather improve health according to grounded health and wellness realities [128] in LMIC.

### Implications

Digital health as a humanitarian modality of assistance has potential but the digital divide factors cannot be overstated as risks that may exclude and discrimination against certain groups. Formative digital health research has been carried out, but this field remains in its infancy. Research into the potential of improving health outcomes remains a crucial gap and more evidence is needed in this regard. Existing scholarship remains dominated by HIC narratives and may not truly represent LMIC thinking. Current participatory action is limited and under-reported and more attention is required to achieve deeper, representative participatory activities are needed to move past the ‘them’ and ‘us’ positionality. Without this, digital health interventions risk perpetuating techno-colonialism through pervasive biases and injustices. Without participation, digital health tools risk being perceived by their users as irrelevant, culturally insensitive, untrusted, and unused. Participation should be advocated for with donors and embedded within staff consciousness and skill sets.

## Limitations

Though every effort was made to produce a rigorous and comprehensive scoping review, we recognise several limitations within our labours. Firstly, the literature search was challenging as the concept terms ‘digital health’ and ‘localisation’ are not uniformly applied and understood consistently within scholarship. Therefore, searching for these terms alongside their synonyms may have inadvertently omitted relevant literature that used alternative terminology. Additionally, though refugee vocabulary is well articulated through the 1951 Refugee Convention, the definition of IDPs is much less well understood and applied. As a result, it may be applied broadly to include economic migrants, transitionary groups, and others. Whilst every effort was taken to include only forcibly displaced people as a result of crises; it is possible that some literature was included or excluded incorrectly based on the terminology used by the research team.

Secondly, our paper is based upon the presupposition that localisation is a positive phenomenon and is beneficial to displaced groups following crises. Though existing scholarship supports this, localisation remains under-conceptualised and theorised, and we cannot claim our presupposition to be true. Further research should be carried out to characterise further and assess localisation and its desirability within humanitarian crises.

Thirdly, we limited our research scope to displaced populations within LMIC. We did this because this reflects the reality of where displaced populations are primarily located, either internally or internationally, at the time of writing. However, some literature covered interventions within LMIC and HIC and presented data cumulatively. Whilst care was taken to review and analyse only data from LMIC contexts; this was not possible in some cases. As a result, all data may not be wholly representative of LMIC.

Fourthly, a limitation of the scoping study model is that it does not seek to appraise the quality of the interventions included within it. The interventions included in this review varied in size, scale and type. Participant numbers were generally low, and where there was follow-up time, it was often short-term, with much of the data being self-reported, which risks introducing a number of biases. Whilst we recognise that research within humanitarian contexts comes with a range of ethical and practical challenges, it cannot be overlooked that data arising from these interventions may not be wholly reflective of rapidly evolving crisis landscapes, and future research should consider this.

Finally, we recognise discussions of vulnerable groups benefit from their participation at all stages so that they are fully represented. Decolonisation debates support this to shift the crisis narrative away from that of victims and passive aid recipients and reframe them as actors capable of leading their own response. Echoing the UN Secretary General’s words that humanitarian responses should be *“as local as possible and as international as necessary”* [42], we aim, through this paper, to highlight our reflexivity to it. We intend to use our international positionality as a mechanism to make space and draw further resources and recognition for local groups in LMIC to take up this role for themselves.

## Conclusion

This scoping review provides critical insight into how the localisation phenomenon currently manifests as participatory action at the crisis level within digitalised health interventions for displaced people in LMIC, as laid out in scholarly literature. With growing numbers of displaced people globally, addressing their health needs is an important issue, with increasing pressure on humanitarian and health actors to use innovative approaches to address this. The theory of digitalisation to address these health burdens is an exciting prospect, but humanitarian health communities have an ethical imperative to act without causing harm and underpin their approaches with evidence [105]. This review found a conspicuous gap here. Despite this incomplete picture and the nuanced digital dividing factors, the literature did attest to displaced populations' interest and willingness to use digital health tools. However, limited practice did not support these outcomes and further investigations into potential biases is required here.

HIC authorship predominantly constructed the narrative within the reviewed literature, with an absence of reflexivity. This reflects the current power dynamics and structural inequalities within the global humanitarian and health systems. This was exemplified by limited participation in digital health circles, which risks techno-colonialism in maintaining the systemic status quo. The localisation agenda has not yet achieved its paradigm shift of power. Further research is required into its conceptualisation, intersectional spheres, and application within a diverse LMIC landscape to achieve long-lasting outcomes.

With protracted displacement becoming ever more prevalent, UNHCR suggests as many as 31 people are displaced per minute [129], the importance of finding long-term, dynamic solutions to address their health in a meaningful way has become more crucial than ever. To do this, international actors should lean back and make space for and promote participation as one dimension of a broader, intersectional movement to shift power for long-lasting impact.

### Supplementary Information

**Additional file 1**. Search syntax.

**Additional file 2**. PRISMA-ScR flow chart.

**Additional file 3**. Data chart.

**Additional file 4**. Reflexivity statement.

**Additional file 5**. PRISMA checklist.

**Additional file 6**. The Emerging Indicators of the Participation Revolution from the GMI Dimensions of Localisation Framework [45] and the Participation Key Performance Indicators from the NEAR Localisation Performance Measurement Framework [69].

## Abbreviations

A4EP: Alliance for Empowering Partnerships
ALNAP: Active Learning Network for Accountability and Performance
C4C: Charter for Change
CHS: Core Humanitarian Standard
Covid-19: Coronavirus Disease 2019
FGDs: Focus group discussion/s
GMI: Global Mentoring Initiative
GPS: Global Positioning System
HIC: High-income country/ies
HIV/AIDS: Human Immunodeficiency Virus/ Acquired Immunodeficiency Syndrome
HP: Humanitarian Principles
ICRC: International Committee of the Red Cross
IDPs: Internally displaced population/s
IHL: International Humanitarian Law
LIC: Low-income-country/ies
LMIC: Low-and-middle-income-country/ies
MENA: The Middle East and North Africa
NEAR: Network for Empowered Aid Response
NCD: Non-communicable disease/s
PCC: Population, Concept, Context
PRISMA: Preferred Reporting Items for Systematic Reviews and Meta-Analyses
PRISMA-ScR: PRISMA extension for scoping reviews
SDG: Sustainable Development Goals
SMS: Short Messaging System
UMIC: Upper-middle-income country/ies
UN: United Nations
UN OCHA: United Nations Office for the Coordination of Humanitarian Affairs
UNHCR: United Nations High Commission for Refugees
USA: United States of America
WHS: World Humanitarian Summit (2016)

## References

[1] UNHCR. UNHCR: Global displacement hits another record, capping decade-long rising trend 2022 [Available from: https://www.unhcr.org/news/press/2022/6/62a9d2b04/unhcr-global-displacement-hits-record-capping-decade-long-rising-trend.html#:~:text=By%20May%202022%2C%20more%20than,events%20seriously%20disturbing%20public%20order.

[2] Larkin HD (2022). Humanitarian need jumps in 2022. JAMA.

[3] Schmid B, Raju E, Kickbusch I, Ganten D, Moeti M (2021). Humanitarian Crisis and Complex Emergencies: Burden of Disease, Response, and Opportunities for Global Health. Handbook of Global Health.

[4] Cantor D, Swartz J, Roberts B, Abbara A, Ager A, Bhutta ZA (2021). Understanding the health needs of internally displaced persons: a scoping review. J Migr. Health.

[5] Fanelli S, Salvatore FP, De Pascale G, Faccilongo N (2020). Insights for the future of health system partnerships in low- and middle-income countries: a systematic literature review. BMC Health Serv Res.

[6] Mills A (2014). Health care systems in low- and middle-income countries. N Engl J Med.

[7] Roberts B, Odong VN, Browne J, Ocaka KF, Geissler W, Sondorp E (2009). An exploration of social determinants of health amongst internally displaced persons in northern Uganda. Confl Heal.

[8] Gostin LO, Roberts AE (2015). Forced migration: the human face of a health crisis. JAMA.

[9] Jacobs C, Ferreira, F. Policies should better support people trapped in long-term refugee situations. UK: The Conversation; 2020 [updated 11 Sep 2020; cited 2021 3 August]. Available from: https://reliefweb.int/report/world/policies-should-better-support-people-trapped-long-term-refugee-situations.

[10] MOAS. The Issues that come from Protracted Displacement n.d. [15 July 2022]. Available from: https://www.moas.eu/blog-the-issues-that-come-from-protracted-displacement/#:~:text=In%20fact%2C%20according%20to%20UNHCR,of%20an%20astounding%2020%20years.

[11] UNHCR. The 1951 Refugee Convention n.d. [18 July 2022]. Available from: https://www.unhcr.org/1951-refugee-convention.html.

[12] Médecins Sans Frontières. The Practical Guide to Humanitarian Law n.d. [18 July 2022]. Available from: https://guide-humanitarian-law.org/content/article/3/humanitarian-principles/.

[13] Koch A, Kuhnt, J. Migration and the 2030 agenda: making everyone count - migrants and refugees in the sustainable development goals2022 18 July 2022]. Available from: https://www.idos-research.de/en/briefing-paper/article/migration-and-the-2030-agenda-making-everyone-count-migrants-and-refugees-in-the-sustainable-development-goals/.

[14] Nahmias P, Krynsky Baal, N. Leaving no (refugee) behind: the new indicator on refugees in the SDG indicator framework is a game-changer 2019 18 July 2022]. Available from: https://www.jips.org/news/10-iaeg-new-indicator-on-refugees-sdg-indicator-framework-a-game-changer/.

[15] European Parliament Research Service (EPRS). The global compact on refugees: Briefing paper 2019 [18 July 2022]. Available from: https://www.europarl.europa.eu/RegData/etudes/BRIE/2018/623550/EPRS_BRI(2018)623550_EN.pdf.

[16] Govt, Germany. Global Compact on Refugees – taking responsibility and sharing the burden 2019 [18 July 2022]. Available from: https://reliefweb.int/report/germany/global-compact-refugees-taking-responsibility-and-sharing-burden.

[17] UNHCR. The Sustainable Development Goals and the Global Compact on Refugees n.d. 18 July 2022]. Available from: https://www.unhcr.org/5efcb5004.pdf.

[18] OECD. Lives in Crises : What Do People Tell Us About the Humanitarian Aid They Receive? 2. Humanitarian assistance leaves some of the most vulnerable behind 2019 [18 July 2022]. Available from: https://www.oecd-ilibrary.org/sites/81e5965c-en/index.html?itemId=/content/component/81e5965c-en.

[19] United Nations. Meetings Coverage: Funding Not Enough to Meet Rising Humanitarian Needs in Syria, Top Official Tells Security Council, as Members Diverge Over Delivery Methods 2022 [18 July 2022]. Available from: https://press.un.org/en/2022/sc14897.doc.htm.

[20] Akhmatova D-M, Akhmatova M-S (2020). Promoting digital humanitarian action in protecting human rights: hope or hype. J. Int. Human. Action.

[21] Mesmar S, Talhouk R, Akik C, Olivier P, Elhajj IH, Elbassuoni S (2016). The impact of digital technology on health of populations affected by humanitarian crises: recent innovations and current gaps. J Public Health Policy.

[22] Bryant. J. Digital technologies and inclusion in humanitarian response 2022 [18 July 2022]. Available from: https://odi.org/en/publications/digital-technologies-and-inclusion-in-humanitarian-response/.

[23] Ronquillo Y, Meyers A., Korvek SJ. Digital Health 2022 [18 July 2022]. Available from: https://www.ncbi.nlm.nih.gov/books/NBK470260/.

[24] UNICEF. UNICEF’s Approach to Digital Health 2018 [18 July 2022]. Available from: https://www.unicef.org/innovation/media/506/file/UNICEF%27s%20Approach%20to%20Digital%20Health%E2%80%8B%E2%80%8B.pdf.

[25] Wilson D, Sheikh A, Görgens M, Ward K (2021). Technology and universal health coverage: examining the role of digital health. J Glob Health.

[26] Arendt-Cassetta L. From digital promise to frontline practice: new and emerging technologies in humanitarian action [Online Report]. Geneva: OCHA; 2021 [cited 2021 4 October]. Available from: https://www.alnap.org/help-library/from-digital-promise-to-frontline-practice-new-and-emerging-technologies-in.

[27] ICRC. Advisory Service: What is International Humanitarian Law?2004 [cited 2023. Available from: https://www.icrc.org/en/doc/assets/files/other/what_is_ihl.pdf.

[28] OCHA. OCHA on Message: Humanitarian Principles2022 [cited 2023. Available from: https://www.unocha.org/sites/unocha/files/OOM_Humanitarian%20Principles_Eng.pdf.

[29] SPHERE. The Sphere Handbook: Humanitarian Charter and Minimum Standards in Humanitarian Response | Fourth Edition [Online Report]. Geneva: Sphere Association; 2018 [cited 2021 18 October]. Available from: https://www.alnap.org/help-library/the-sphere-handbook-humanitarian-charter-and-minimum-standards-in-humanitarian-response.

[30] CHS Alliance. CHS Guidance Notes and Indicators [Online Report]. Switzerland: CHS Alliance; 2019 [cited 2021 18 October]. Available from: https://aap-inclusion-psea.alnap.org/help-library/chs-guidance-notes-and-indicators-0.

[31] OCHA. Global Humanitarian Overview 2022 2022 [18 July 2022]. Available from: https://gho.unocha.org/delivering-better/local-actors-play-key-role-humanitarian-action#:~:text=Local%20actors%20play%20a%20key%20role%20as%20first%20responders%20in,responses%20at%20the%20country%20level.

[32] McGoldrick C. Humanitarianism at breaking point? New roles for local and international actors 2016 [18 July 2022]. Available from: https://blogs.icrc.org/law-and-policy/2016/08/19/humanitarianism-local-international-actors/.

[33] Ward P (2020). Capitalising on ‘local knowledge’: the labour practices behind successful aid projects—the case of Jordan. Curr Sociol.

[34] Telford J, Cosgrave J (2007). The international humanitarian system and the 2004 Indian Ocean earthquake and tsunamis. Disasters.

[35] Macrae G (2008). Could the system work better? Scale and local knowledge in humanitarian relief. Dev Pract.

[36] Robillard S, Atim, T., Maxwell, D.,. Localization: A “Landscape” Report 2021 [18 July 2022]. Available from: https://fic.tufts.edu/publication-item/localization-a-landscape-report/.

[37] Bennett C, Foley, M. and Pantuliano, S. Time to let go: A three-point proposal to change the humanitarian system 2016 [18 July 2022]. Available from: https://www.alnap.org/help-library/time-to-let-go-a-three-point-proposal-to-change-the-humanitarian-system.

[38] Barbelet. V., Davies. G., Flint. J., Davey. E. Interrogating the evidence base on humanitarian localisation 2021 [18 July 2022]. Available from: https://cdn.odi.org/media/documents/Localisation_lit_review_WEB.pdf.

[39] OCHA. World Humanitarian Summit: Commitments to action [Online Report]. Turkey: OCHA; 2016 [cited 2021 27 October]. Available from: https://www.alnap.org/help-library/world-humanitarian-summit-commitments-to-action.

[40] Agenda for Humanity. World Humanitarian Summit 2016 2016. Available from: https://agendaforhumanity.org/summit.

[41] Canyon D, The BF (2016). World Humanitarian summit report card: both failing marks and substantive gains for an increasingly globalized humanitarian landscape. PLoS Curr.

[42] United Nations. Secretary-General, at Round Table, Commits to Making Humanitarian Action ‘Local as Possible, International as Necessary’ 2016 [18 July 2022]. Available from: https://press.un.org/en/2016/sgsm17778.doc.htm.

[43] IASC. A participation revolution: include people receiving aid in making the decisions which affect their lives 2016 [18 July 2022]. Available from: https://interagencystandingcommittee.org/a-participation-revolution-include-people-receiving-aid-in-making-the-decisions-which-affect-their-lives.

[44] Fiori J, Espada F, Field J, Dicker S. The echo chamber: results, management and the humanitarian effectiveness Agenda 2016 [18 July 2022]. Available from: https://bura.brunel.ac.uk/bitstream/2438/20041/1/FullText.pdf.

[45] Van Brabant. K., Patel. S. Localisation in practice: emerging indicators & practical recommendations [Report]. Washington DC: Global Mentoring Initiative; 2018 [cited 2021 3 August]. Available from: https://reliefweb.int/sites/reliefweb.int/files/resources/Localisation-In-Practice-Full-Report-v4.pdf.

[46] A4EP, ECOWEB, OCHA. Moving forward localisation of humanitarian action in the Philippines: empowerment leads to better humanitarian outcomes, February–July 2021 2021 [18 July 2022]. Available from: https://reliefweb.int/report/philippines/moving-forward-localisation-humanitarian-action-philippines-empowerment-leads.

[47] DuBois M. The new humanitarian basics 2018 [18 July 2022]. Available from: https://cdn.odi.org/media/documents/12201.pdf.

[48] Humanitarian Leadership Academy. Unpacking Localization [Report]. Humanitarian Leadership Academy; 2019 [updated October 2019; cited 2021 8 August]. Available from: https://www.humanitarianleadershipacademy.org/pdf/unpacking-localization/.

[49] O’Connor S, Hanlon P, O’Donnell CA, Garcia S, Glanville J, Mair FS (2016). Understanding factors affecting patient and public engagement and recruitment to digital health interventions: a systematic review of qualitative studies. BMC Med Inform Decis Mak.

[50] Gearing RE, Schwalbe CS, MacKenzie MJ, Brewer KB, Ibrahim RW, Olimat HS (2012). Adaptation and translation of mental health interventions in Middle Eastern Arab countries: a systematic review of barriers to and strategies for effective treatment implementation. Int J Soc Psychiatry.

[51] Owen E, Massazza A, Roberts B, Lokot M, Fuhr DC (2022). "Nothing about us, without us"? A qualitative study of service user involvement in the development of lay-delivered psychological interventions in contexts affected by humanitarian crises. J Migr Health.

[52] Greenhalgh T, Hinton L, Finlay T, Macfarlane A, Fahy N, Clyde B (2019). Frameworks for supporting patient and public involvement in research: Systematic review and co-design pilot. Health Expect.

[53] Arnstein SR (1969). A ladder of citizen participation. J Am Inst Plann.

[54] Network C. How-To Guide on Collective Communication and Community Engagement in humanitarian actionn.d. [cited 2023. Available from: https://www.cdacnetwork.org/tools-guidance/how-to-guide-on-collective-communication-and-community-engagement-in-humanitarian-action.

[55] OCHA. OCHA on Message: Accountability to Affected People2023 [cited 2023. Available from: https://www.unocha.org/sites/unocha/files/OCHA%20on%20Message%20Accountability%20to%20Affected%20People.pdf.

[56] ALNAP. Engagement of crisis-affected people in humanitarian action: Background Paper2014 [cited 2023. Available from: https://sohs.alnap.org/system/files/content/resource/files/main/background-paper-29th-meeting.pdf.

[57] ALNAP. Participation By Crisis - Affected Populations in Humanitarian Action: A Handbook for Practitionersn.d. [cited 2023. Available from: https://www.humanitarianlibrary.org/resource/participation-crisis-affected-populations-humanitarian-action-handbook-practitioners-0.

[58] British Red Cross. Is aid really changing? 2021 [18 July 2022]. Available from: https://www.redcross.org.uk/-/media/documents/humanitarian/reportis-aid-really-changing-what-the-covid19-response-tells-us-about--localisation-decolonisation-a.pdf.

[59] Aloudat T, Khan T (2022). Decolonising humanitarianism or humanitarian aid?. PLOS Global Public Health.

[60] O’Sullivan K, Clark S, Marshall K, MacLachlan M (2021). A just digital framework to ensure equitable achievement of the Sustainable Development Goals. Nat Commun.

[61] Townsend A, McMahon M (2021). COVID-19 and BLM: humanitarian contexts necessitating principles from first nations world views in an intercultural social work curriculum. Br J Soc Work.

[62] Simonson J (2021). Police reform through a power lens. Yale Law J.

[63] Charter4Change. Charter for Change: Localisation of Humanitarian Aid n.d. [18 July 2022]. Available from: https://charter4change.org/.

[64] Near NGO. Near n.d. [18 July 2022]. Available from: https://www.near.ngo/.

[65] A4EP. Localisation: what does it mean? 2022 [18 July 2022]. Available from: https://a4ep.net/?p=1438.

[66] Global Refugee Network. Centering Refugees in Global Refugee Response n.d. [18 July 2022]. Available from: https://www.globalrefugeenetwork.org/.

[67] Sabatier PA (1986). Top-down and bottom-up approaches to implementation research: a critical analysis and suggested synthesis. J Publ Policy.

[68] Gibbons P, Otieku-Boadu C. The Question is not “If to Localise?” but Rather “How to Localise?”: Perspectives from Irish Humanitarian INGOs. Frontiers in Political Science. 2021;3.

[69] NEAR NGO. Localization performance measurement framework (LPMF) 2019 [18 July 2022]. Available from: https://www.near.ngo/lpmf.

[70] Federal Foreign Office. Fundamental principles of humanitarian assistance n.d. [18 July 2022]. Available from: https://www.auswaertiges-amt.de/en/aussenpolitik/themen/humanitarianassistance/-/256630.

[71] Benson J. Scoping Review of Digital Health Approaches for Displaced People in Low Resource Settings: Protocol 2021 [18 July 2022]. Available from: https://osf.io/9d25r.

[72] Aromataris E. Munn Z. JBI Manual for Evidence Synthesis [Internet]. JBI; 2020 [updated 2021; cited 2021 23 June]. Available from: https://synthesismanual.jbi.global. 10.46658/JBIMES-20-01.

[73] ICRC. International Committee of the Red Cross Resource Centre [Website]. ICRC; n.d. [cited 2022 2 April]. Available from: https://www.icrc.org/en/resource-centre.

[74] Oxfam. All of Oxfam Digital Repository [Website]. Oxfam; n.d [cited 2022 2 April]. Available from: https://oxfamilibrary.openrepository.com/.

[75] ALNAP. HELP Library [Webpage]. ALNAP; n.d. [cited 2022 2 April]. Available from: https://www.alnap.org/help-library.

[76] Cochrane. LMIC Filters [Website]. Cochrane; 2020 [cited 2022 2 April]. Available from: https://epoc.cochrane.org/lmic-filters.

[77] Arksey H, O'Malley L (2005). Scoping studies: towards a methodological framework. Int J Soc Res Methodol.

[78] Page MJ, McKenzie JE, Bossuyt PM, Boutron I, Hoffmann TC, Mulrow CD (2021). The PRISMA 2020 statement: an updated guideline for reporting systematic reviews. BMJ.

[79] Foucault M. The archaeology of knowledge and discourse on language 1972 [18 July 2022]. Available from: https://monoskop.org/images/9/90/Foucault_Michel_Archaeology_of_Knowledge.pdf.

[80] Mbuya Isaac G. MunIo. Critical systems thinking, theory and practice: A case study of an intervention In two British local authorities: University of Hull; 1997.

[81] Flood RL, Romm NRA (1996). Plurality revisited: diversity management and triple loop learning. Syst Pract.

[82] Gioia DA, Pitre E (1990). Multiparadigm perspectives on theory building. Acad Manag Rev.

[83] Weaver GR, Gioia DA (1994). Paradigms lost: incommensurability vs structurationist inquiry. Organ Stud.

[84] Molinengo G. Flows of power: an analytical framework for the study of collaboration. Critical Policy Studies. 2022:1–22.

[85] Topp SM, Schaaf M, Sriram V, Scott K, Dalglish SL, Nelson EM (2021). Power analysis in health policy and systems research: a guide to research conceptualisation. BMJ Glob Health.

[86] Hilsdon J, Sentito, E., Dawson, J., Gentle, C., Allison, J. Critical thinking. Learning Development Study Guide 8. Plymouth: University of Plymouth 2010 [18 July 2022]. Available from: https://www.plymouth.ac.uk/uploads/production/document/path/1/1710/Critical_Thinking.pdf.

[87] Hilsdon J, Bitzer, EM. . To become an asker of questions: a ‘functional-narrative’ model to assist students in preparing post-graduate research proposals. South Afr J Higher Educ. 2007;21(8):1191–203.

[88] UNHCR. Global Trends: Global Forced Displacement 2021 [18 July 2022]. Available from: https://www.unhcr.org/globaltrends.html.

[89] Augusterfer EF, Mollica RF, Lavelle J (2015). A review of telemental health in international and post-disaster settings. Int Rev Psychiatry.

[90] El-Khatib Z, El-Halabi S, Abu Khdeir M, Khader YS (2020). Children Immunization App (CImA), low-cost digital solution for supporting Syrian Refugees in Zaatari Camp in Jordan - General Description. Stud Health Technol Inform.

[91] Narla NP, Surmeli A, Kivlehan SM (2020). Agile application of digital health interventions during the COVID-19 refugee response. Ann Glob Health.

[92] Nasir S, Goto R, Kitamura A, Alafeef S, Ballout G, Hababeh M (2020). Dissemination and implementation of the e-MCH H andbook, UNRWA's newly released maternal and child health mobile application: a cross-sectional study. BMJ Open.

[93] Burchert S, Alkneme MS, Bird M, Carswell K, Cuijpers P, Hansen P, et al. User-centered app adaptation of a low-intensity E-mental health intervention for syrian refugees. Front Psychiatry. 2019;9(663). 10.3389/fpsyt.2018.00663 PMC6355704 30740065

[94] Heim E, Ramia JA, Hana RA, Burchert S, Carswell K, Cornelisz I (2021). Step-by-step: feasibility randomised controlled trial of a mobile-based intervention for depression among populations affected by adversity in Lebanon. Internet Interv.

[95] Tomita A, Kandolo KM, Susser E, Burns JK (2016). Use of short messaging services to assess depressive symptoms among refugees in South Africa: implications for social services providing mental health care in resource-poor settings. J Telemed Telecare.

[96] Saleh S, Farah A, El Arnaout N, Dimassi H, El Morr C, Muntaner C (2018). mHealth use for non-communicable diseases care in primary health: patients' perspective from rural settings and refugee camps. J Public Health.

[97] M. C, Herson M. Forced Migration Review: Armed non-state actors and displacement [Online Report]. Oxford, UK: Refugee Studies Centre; 2011 [cited 2021 8 November]. Available from: https://www.alnap.org/help-library/forced-migration-review-armed-non-state-actors-and-displacement.

[98] Ben-Zeev D, Fathy C, Jonathan G, Abuharb B, Brian RM, Kesbeh L (2017). mHealth for mental health in the Middle East: need, technology use, and readiness among Palestinians in the West Bank. Asian J Psychiatr.

[99] Soron TR, Heanoy EZ, Udayasankaran JG (2019). Did Bangladesh miss the opportunity to use telepsychiatry in the rohingya refugee crisis?. Lancet Psychiatry.

[100] Ashfaq A, Esmaili S, Najjar M, Batool F, Mukatash T, Al-Ani HA (2020). Utilization of mobile mental health services among syrian refugees and other vulnerable arab populations: a systematic review. Int J Environ Res Public Health.

[101] Jefee-Bahloul H (2014). Use of telepsychiatry in areas of conflict: the Syrian refugee crisis as an example. J Telemed Telecare.

[102] Nassan M, Frye MA, Adi A, Alarcon RD (2015). Telepsychiatry for post-traumatic stress disorder: a call for action in the Syrian conflict. Lancet Psychiatry.

[103] Yousef H, Al-Sheyab N, Al Nsour M, Khader Y, Al Kattan M, Bardus M (2021). Perceptions toward the use of digital technology for enhancing family planning services: focus group discussion with beneficiaries and key informative interview with Midwives. J Med Internet Res.

[104] Ndubuisi NE (2021). Noncommunicable diseases prevention in low- and middle-income countries: an overview of health in all policies (HiAP). Inquiry.

[105] Perakslis ED (2018). Using digital health to enable ethical health research in conflict and other humanitarian settings. Confl Heal.

[106] International Federation of Red Cross and Red Crescent Societies. World Disasters Report 2013 - Focus on technology and the future of humanitarian action [Online Report]. Geneva: IFRC; 2013 [cited 2021 2 November]. Available from: https://www.alnap.org/help-library/world-disasters-report-2013-focus-on-technology-and-the-future-of-humanitarian-action.

[107] Yankah E, Mohamed O, Wringe A, Afaneh O, Saleh M, Speed O (2020). Feasibility and acceptability of mobile phone platforms to deliver interventions to address gender-based violence among Syrian adolescent girls and young women in Izmir, Turkey. Vulnerable Child Youth Stud.

[108] Talhouk R, Akik C, Araujo-Soares V, Ahmad B, Mesmar S, Olivier P (2020). Integrating health technologies in Health Services for Syrian Refugees in Lebanon: Qualitative Study. J Med Internet Res.

[109] Samuel Hall. Innovating mobile solutions for refugees in East Africa [Online Report]. Unknown: Samuel Hall; 2021 [cited 2021 20 October]. Available from: https://www.alnap.org/help-library/innovating-mobile-solutions-for-refugees-in-east-africa.

[110] Syria Independent Monitoring (SIM) team. The use of mobile technology for humanitarian programming in Syria: potential and constraints [Online Report]. UK: UK Foreign, Commonwealth and Development Office; 2017 [cited 2021 26 October]. Available from: https://www.alnap.org/help-library/the-use-of-mobile-technology-for-humanitarian-programming-in-syria-potential-and-0.

[111] Forced Migration Review. Forced Migration Review: The Technology Issue [Online Report]. Oxford, UK: Refugee Studies Centre; 2011 [cited 2021 8 November]. Available from: https://www.alnap.org/help-library/forced-migration-review-the-technology-issue.

[112] Hallow D, Mitchell J, Gladwell C, Aggiss R. Mobile Technology in Emergencies: Efficient cash transfer mechanisms and effective two-way communication with disaster-affected communities using mobile phone technology [Online Report]. UK: Save the Children; 2012 [cited 2021 3 November]. Available from: https://www.alnap.org/help-library/mobile-technology-in-emergencies-efficient-cash-transfer-mechanisms-and-effective-two.

[113] The World Bank. The World by Income and Region 2021 [18 July 2022]. Available from: https://datatopics.worldbank.org/world-development-indicators/the-world-by-income-and-region.html.

[114] Stark AL, Geukes C, Dockweiler C (2022). Digital health promotion and prevention in settings: scoping review. J Med Internet Res.

[115] Maitland. C. Lost: Syrian refugees and the information gap: Internews; 2013 [19 July 2022]. Available from: https://www.internews.org/sites/default/files/resources/Internews_Lost_SyriaReport_Nov2013_web.pdf

[116] OECD. Glossary of Statistical Terms: Digital Divide 2002 [19 July 2022]. Available from: https://stats.oecd.org/glossary/detail.asp?ID=4719.

[117] United Nations. Digital Divide ‘a Matter of Life and Death’ amid COVID-19 Crisis, Secretary‑General Warns Virtual Meeting, Stressing Universal Connectivity Key for Health, Development 2021 [19 July 2022]. Available from: https://press.un.org/en/2020/sgsm20118.doc.htm.

[118] Fast L, Bennett C. From the ground up: it’s about time for local humanitarian action 2020 [19 July 2022]. Available from: https://odi.org/en/publications/from-the-ground-up-its-about-time-for-local-humanitarian-action/.

[119] Barakat S, Milton S (2020). Localisation across the humanitarian-development-peace nexus. J Peacebuilding Dev.

[120] Barbelet V, Davies, G., Flint, J. and Davey, E,. Interrogating the evidence base on humanitarian localisation [Report]. UK: Overseas Development Institute; 2021 [updated June 2021; cited 2021 8 August]. Available from: https://odi.org/en/publications/interrogating-the-evidence-base-on-humanitarian-localisation-a-literature-study/.

[121] Anderson M, Brown, D., Jean, I. Time to Listen: Hearing People on the Receiving End of International Aid 2012 [18 July 2022]. Available from: https://www.cdacollaborative.org/publication/time-to-listen-hearing-people-on-the-receiving-end-of-international-aid/.

[122] Ormel I, Salsberg J, Hunt M, Doucet A, Hinton L, Macaulay AC, et al. Key issues for participatory research in the design and implementation of humanitarian assistance: a scoping review. Glob Health Action. 2020;13(1):1826730-. 10.1080/16549716.2020.1826730 PMC7594848 33073736

[123] Shuayb M. Localisation only pays lip service to fixing aid’s colonial legacy 2022 [18 July 2022]. Available from: https://www.thenewhumanitarian.org/opinion/2022/2/8/Localisation-lip-service-fixing-aid-colonial-legacy.

[124] Bowsher G, El Achi N, Augustin K, Meagher K, Ekzayez A, Roberts B (2021). eHealth for service delivery in conflict: a narrative review of the application of eHealth technologies in contemporary conflict settings. Health Policy Plan.

[125] Sandvik K, Lohne, K., in Rejali, S., Heiniger, Y. The role of digital technologies in humanitarian law, policy and action: Charting a path forward 2021 [18 July 2022]. Available from: https://international-review.icrc.org/articles/digital-technologies-humanitarian-law-policy-action-913#footnoteref48_jnb7clk.

[126] PHAP. Event report: Participation in Practice: Examples of inclusive action for a “Participation Revolution”2020. Available from: https://phap.org/common/Uploaded%20files/Webinar%20documents/200326%20-%20SCHR%20GB%20Workstream%206%20-%20Report.pdf.

[127] Madianou M. Technocolonialism: Digital Innovation and Data Practices in the Humanitarian Response to Refugee Crises. Social Media + Society. 2019;5(3):2056305119863146.

[128] Duclos D, Ekzayez A, Ghaddar F, Checchi F, Blanchet K (2019). Localisation and cross-border assistance to deliver humanitarian health services in North-West Syria: a qualitative inquiry for the Lancet-AUB Commission on Syria. Confl Heal.

[129] UNHCR. Global Trends: Forced displacement in 2017 2017 [19 July 2022]. Available from: https://www.unhcr.org/globaltrends2017/.

[130] World Bank. World Bank Country and Lending Groups [Website]. World Bank Group; 2021 [cited 2022 2 April]. Available from: https://datahelpdesk.worldbank.org/knowledgebase/articles/906519-world-bank-country-and-lending-groups#:~:text=%EF%BB%BF%EF%BB%BF%20For%20the%20current,those%20with%20a%20GNI%20per.

[131] Financing SSPoHSa, Coordinator AFP, Coordinator NEARO, Refugee Health P, Professor HDA, Coordinator CEMAP, et al. mHealth use for non-communicable diseases care in primary health: patients' perspective from rural settings and refugee camps. J Public Health. 2018;40:ii52–ii63. 10.1093/pubmed/fdy172 PMC6294037 30307516

[132] Saleh S, El Arnaout N, Faulkner JR, Sayegh MH (2019). Sijilli: a mobile electronic health records system for refugees in low-resource settings. Lancet Glob Health.

[133] Brophy-Williams S, Hardman, J., Leaning, J., Meier, P.,, Olafsson G, Pham, P., Quintanilla, J., Bergtora Sandvik, K., and Segaren, N. World Disasters Report: Focus on technology and the future of humanitarian action [Report]. Geneva: International Federation of Red Cross and Red Crescent Societies; 2013 [cited 2021 3 August]. 1–283]. Available from: https://www.ifrc.org/PageFiles/134658/WDR%202013%20complete.pdf.

[134] Martin A. Displaced and Disconnected [Online Report]. Unknown: UNHCR; 2019 [cited 2021 18 October]. Available from: https://www.alnap.org/help-library/displaced-and-disconnected-0.

[135] UNHCR. Connecting Refugees: How Internet and Mobile Connectivity can Improve Refugee Well-Being and Transform Humanitarian Action [Online Report]. Geneva: UNHCR; 2016 [cited 2021 27 October]. Available from: https://www.alnap.org/help-library/connecting-refugees-how-internet-and-mobile-connectivity-can-improve-refugee-well-being.

[136] Alliance Development Works. World Risk Report 2012 [Online Report]. Berlin, Germany: Bündnis Entwicklung Hilft Alliance Development Works; 2012 [cited 2021 3 November]. Available from: https://www.alnap.org/help-library/world-risk-report-2012.

[137] Financing SSPoHSa, Coordinator AFP, Coordinator NEAROaRHP, Professor HDA, Coordinator CEMAPaHIC, Professor CM, et al. mHealth use for non-communicable diseases care in primary health: patients' perspective from rural settings and refugee camps. Journal of Public Health. 2018;40:ii52-ii63. 10.1093/pubmed/fdy172 PMC6294037 30307516

[138] Khader YS, Laflamme L, Schmid D, El-Halabi S, Abu Khdair M, Sengoelge M (2019). Children Immunization App (CImA) Among Syrian Refugees in Zaatari Camp, Jordan: Protocol for a Cluster Randomized Controlled Pilot Trial Intervention Study. JMIR Res Protoc.

