# Supplementary material for: Localisation of digital health tools used by displaced populations in low and middle-income settings: a scoping review and critical analysis of the Participation Revolution
Source: Confl Health. 2023 Apr 15;17:20. doi: 10.1186/s13031-023-00518-9 (PMC10105546; doi:10.1186/s13031-023-00518-9)
Supplement: Supplementary file 1 — Additional file 1. Search syntax. [file 13031_2023_518_MOESM1_ESM.docx]

**Supporting Materials 1: Search syntaxes**

1. CINAHL via EBSCO:

| **PCC** | **Keywords and Subject Terms** |
| --- | --- |
| Population | "Aid Recipient*" OR "Asylum Seek*" OR Beneficiar* OR "conflict affected person*" OR "conflict affected population*" OR "disaster victim*" OR "displaced population*" OR "displaced people*" OR "displaced person*" Or "internally displaced" OR "internally displaced population*" OR "internally displaced people*" OR "internally displaced person*" OR evacuat* OR "forcibly displace*" OR "forcibly displaced population*" OR "forcibly displaced people*" OR "forcibly displaced person*" OR "political asylum seek*" OR "political refugee*" OR refugee* OR rescue* OR victim* |
| Population Subject terms | MH "Refugees+" |
| Concept 1 | computer* OR "computer-based" OR "cyber medicine*" OR "digital electronic*" OR "digital technolog*" OR ehealth OR e-health OR "e health" OR "expert system*" OR "health 2.0" OR "mobile health" OR internet OR mhealth OR m-health OR "m health" OR mobile* OR "mobile technologies*" OR "digital health" OR SMS OR "text message*" OR telehealth OR telemedicine OR "web health" OR "smart phone*" OR laptop* OR tablet* OR app OR apps |
| Concept 1 Subject terms | MH "Digital Technology+" OR MH "Expert Systems" OR MH "Telemedicine+" OR MH "Smartphone" OR MH "Mobile Applications" |
| Concept 2 | accountabilit* OR "social accountabilit*" OR "community action*" OR "patient activation*" OR adapt* OR communitarianism OR "community action*" OR "community involvement*" OR "consumer involvement*" OR "consumer participation*" OR "patient empowerment*" OR empowerment* OR engagement* OR "patient engagement*" OR "stakeholder engagement*" OR feedback* OR individualis* OR individualiz* OR "community involvement*" OR "user engagement*" OR "social obligation*" OR "obligations societ*" OR "patient participation rate*" OR "community participation" OR "patient participation" OR "patient activation" OR "patient participation rate"* OR personalis* OR personaliz* OR "public participation" OR "social responsibilit*" OR "social accountabilit*" OR "social behaviour*" OR "social obligation*" OR localisation OR localised OR localized OR localization OR "stakeholder engagement*" OR "stakeholder role*" OR tailor* OR "patient participation" OR "participatory appraisal*" OR "emancipatory intervention*" OR empowerment* OR "collaborative inquir*" OR "collaborative enquir*" OR "social reconnaissance" OR "community-based participatory intervention*" OR "community-based participatory action*" OR "user-centered" OR "user-designed" OR "user-led" OR "user centred" OR "user designed" OR "user led" OR "user-centered" OR "user centered" OR "accountability affected people" OR "accountability affected person*" OR "accountability affected populations" OR "beneficiary engagement" OR "person-centred" OR "person centred" OR "person-centered" OR "person centered" OR "social behavior*" |
| Concept 2 Subject terms | MH "Social Responsibility+" OR MH "Consumer Participation" OR MH "Stakeholder Participation" OR MH "Social Behavior" OR MH Empowerment |
| Context | "absolute povert*" OR "developing countr*" OR "extreme povert*" OR indigenc* OR indigent* OR LIC OR LMIC OR "low GDP" OR "low GNP" OR "low gross domestic product" OR "low gross national product" OR "low resource*" OR "low income" OR "low income population*" OR "low resource population*" OR MIC OR "middle income*" OR "middle income countr*" OR "middle resource*" OR poverty* OR "third world" OR "3rd world" OR "transitional context*" OR "transitional nation*" OR "transitional countr*" OR "transitional state*" OR "under-developed" OR "under-developing" OR "under developed" OR "under developing" OR underdeveloped OR "low income econom*" OR "low middle income econom*" OR Africa OR Asia OR Caribbean OR "West Indies" OR "South America" OR "Latin America" OR "Central America" OR underserved OR "under served" OR under-served OR poor OR deprived OR colonised OR colonized |
| Cochrane LMIC filter | Afghanistan OR Albania OR Algeria OR Angola OR Armenia OR Armenian OR Azerbaijan OR Bangladesh OR Benin OR Byelarus OR Byelorussian OR Belarus OR Belorussian OR Belorussia OR Belize OR Bhutan OR Bolivia OR Bosnia OR Herzegovina OR Hercegovina OR Botswana OR Brasil OR Brazil OR Bulgaria OR "Burkina Faso" OR "Burkina Fasso" OR Burundi OR Urundi OR Cambodia OR "Khmer Republic" OR Cameroon OR Cameroons OR Cameron OR Camerons OR "Cape Verde" OR "Central African Republic" OR Chad OR China OR Colombia OR Comoros OR "Comoro Islands" OR Comores OR Congo OR Zaire OR "Costa Rica" OR "Cote d’Ivoire" OR "Ivory Coast" OR Cuba OR Djibouti OR Dominica OR "Dominican Republic" OR "East Timor" OR "East Timur" OR "Timor Leste" OR Ecuador OR Egypt OR "United Arab Republic" OR "El Salvador" OR Eritrea OR Ethiopia OR Fiji OR Gabon OR "Gabonese Republic" OR Gambia OR Gaza OR "Georgia Republic" OR "Georgian Republic" OR Ghana OR Grenada OR Guatemala OR Guinea OR Guiana OR Guyana OR Haiti OR Honduras OR India OR Maldives OR Indonesia OR Iran OR Iraq OR Jamaica OR Jordan OR Kazakhstan OR Kazakh OR Kenya OR Kiribati OR Korea OR Kosovo OR Kyrgyzstan OR Kirghizia OR "Kyrgyz Republic" OR Kirghiz OR Kirgizstan OR "Lao PDR" OR Laos OR Lebanon OR Lesotho OR Liberia OR Libya OR Macedonia OR Madagascar OR "Malagasy Republic" OR Malaysia OR "Malaya Sabah" OR Sarawak OR Malawi OR Nyasaland OR Mali OR "Marshall Islands" OR Mauritania OR Mauritius OR "Agalega Islands" OR Mexico OR Micronesia OR Moldova OR Moldovia OR Moldovian OR Mongolia OR Montenegro OR Morocco OR Mozambique OR Myanmar OR Myanma OR Burma OR Namibia OR Nepal OR Nicaragua OR Niger OR Nigeria OR Pakistan OR Paraguay OR Peru OR Philippines OR Philipines OR Phillipines OR Phillippines OR Romania OR Rumania OR Roumania OR Russia OR Russian OR Rwanda OR Ruanda OR "Saint Lucia" OR "St Lucia" OR "Saint Vincent" OR "St Vincent" OR Grenadines OR Samoa OR "Samoan Islands" OR "Sao Tome" OR Senegal OR Serbia OR Montenegro OR "Sierra Leone" OR "Sri Lanka" OR Ceylon OR "Solomon Islands" OR Somalia OR "South Africa" OR Sudan OR Suriname OR Surinam OR Swaziland OR Syria OR Tajikistan OR Tadzhikistan OR Tadjikistan OR Tadzhik OR Tanzania OR Thailand OR Togo OR "Togolese Republic" OR Tonga OR Tunisia OR Turkey OR Turkmenistan OR Turkmen OR Uganda OR Ukraine OR USSR OR "Soviet Union" OR "Union of Soviet Socialist Republics" OR Uzbekistan OR Uzbek OR Vanuatu OR "New Hebrides" OR Venezuela OR Vietnam OR "Viet Nam" OR "West Bank" OR Yemen OR Zambia OR Zimbabwe OR Rhodesia |
| Context Subject terms | MH Poverty+ OR MH "Developing Countries" OR MH "Africa+" OR MH "Asia+" OR MH "West Indies+" OR MH "South America+" OR MH "Latin America" OR MH "Central America+" OR MH "Special Populations" |
| combination A | S1 OR S2 |
| combination B | S3 OR S4 |
| combination C | S5 OR S6 |
| combination D | S7 OR S8 OR S9 |
| combination E | S10 AND S11 AND S12 AND S13 |
| Date filter | 2010 - present |

Search date: 21.9.21

Hits: 62

1. Medline via PubMed:

| **PCC** | **Keywords and MeSH Terms** |
| --- | --- |
| Population | "Aid Recipient*" OR "Asylum Seek*" OR Beneficiar* OR "conflict affected person*" OR "conflict affected population*" OR "disaster victim*" OR "displaced population*" OR "displaced people*" OR "displaced person*" Or "internally displaced" OR "internally displaced population*" OR "internally displaced people*" OR "internally displaced person*" OR evacuat* OR "forcibly displace*" OR "forcibly displaced population*" OR "forcibly displaced people*" OR "forcibly displaced person*" OR "political asylum seek*" OR "political refugee*" OR refugee* OR rescue* OR victim* |
| Population MeSH | "refugees"[MeSH] OR "disaster victims"[MeSH] |
| Concept 1 | computer* OR "computer-based" OR "cyber medicine*" OR "digital electronic*" OR "digital technolog*" OR ehealth OR e-health OR "e health" OR "expert system*" OR "health 2.0" OR "mobile health" OR internet OR mhealth OR m-health OR "m health" OR mobile* OR "mobile technologies*" OR "digital health" OR SMS OR "text message*" OR telehealth OR telemedicine OR "web health" OR "smart phone*" OR laptop* OR tablet* OR app OR apps |
| Concept 1 MeSH | "digital technology"[MeSH] OR "expert systems"[MeSH] OR "telemedicine"[MeSH] OR "smartphone"[MeSH] OR "mobile applications"[MeSH] |
| Concept 2 | accountabilit* OR "social accountabilit*" OR "community action*" OR "patient activation*" OR adapt* OR communitarianism OR "community action*" OR "community involvement*" OR "consumer involvement*" OR "consumer participation*" OR "patient empowerment*" OR empowerment* OR engagement* OR "patient engagement*" OR "stakeholder engagement*" OR feedback* OR individualis* OR individualiz* OR "community involvement*" OR "user engagement*" OR "social obligation*" OR "obligations societ*" OR "patient participation rate*" OR "community participation" OR "patient participation" OR "patient activation" OR "patient participation rate*" OR personalis* OR personaliz* OR "public participation" OR "social responsibilit*" OR "social accountabilit*" OR "social behaviour*" OR "social obligation*" OR localisation OR localised OR localized OR localization OR "stakeholder engagement*" OR "stakeholder role*" OR tailor* OR "patient participation" OR "participatory appraisal*" OR "emancipatory intervention*" OR empowerment* OR "collaborative inquir*" OR "collaborative enquir*" OR "social reconnaissance" OR "community-based participatory intervention*" OR "community-based participatory action*" OR "user-centered" OR "user-designed" OR "user-led" OR "user centred" OR "user designed" OR "user led" OR "user-centered" OR "user centered" OR "accountability affected people" OR "accountability affected person*" OR "accountability affected populations" OR "beneficiary engagement" OR "person-centred" OR "person centred" OR "person-centered" OR "person centered" OR "social behavior*" |
| Concept 2 MeSH | "social responsibility"[MeSH] OR "community participation"[MeSH] OR "patient participation"[MeSH] OR "stakeholder participation"[MeSH] OR "social behavior"[MeSH] OR "empowerment"[MeSH] |
| Context | "absolute povert*" OR "developing countr*" OR "extreme povert*" OR indigenc* OR indigent* OR LIC OR LMIC OR "low GDP" OR "low GNP" OR "low gross domestic product" OR "low gross national product" OR "low resource*" OR "low income" OR "low income population*" OR "low resource population*" OR MIC OR "middle income*" OR "middle income countr*" OR "middle resource*" OR poverty* OR "third world" OR "3rd world" OR "transitional context*" OR "transitional nation*" OR "transitional countr*" OR "transitional state*" OR "under-developed" OR "under-developing" OR "under developed" OR "under developing" OR underdeveloped OR "low income econom*" OR "low middle income econom*" OR Africa OR Asia OR Caribbean OR "West Indies" OR "South America" OR "Latin America" OR "Central America" OR underserved OR "under served" OR under-served OR poor OR deprived OR colonised OR colonized |
| Cochrane LMIC filter | Afghanistan OR Albania OR Algeria OR Angola OR Armenia OR Armenian OR Azerbaijan OR Bangladesh OR Benin OR Byelarus OR Byelorussian OR Belarus OR Belorussian OR Belorussia OR Belize OR Bhutan OR Bolivia OR Bosnia OR Herzegovina OR Hercegovina OR Botswana OR Brasil OR Brazil OR Bulgaria OR "Burkina Faso" OR "Burkina Fasso" OR Burundi OR Urundi OR Cambodia OR "Khmer Republic" OR Cameroon OR Cameroons OR Cameron OR Camerons OR "Cape Verde" OR "Central African Republic" OR Chad OR China OR Colombia OR Comoros OR "Comoro Islands" OR Comores OR Congo OR Zaire OR "Costa Rica" OR "Cote d’Ivoire" OR "Ivory Coast" OR Cuba OR Djibouti OR Dominica OR "Dominican Republic" OR "East Timor" OR "East Timur" OR "Timor Leste" OR Ecuador OR Egypt OR "United Arab Republic" OR "El Salvador" OR Eritrea OR Ethiopia OR Fiji OR Gabon OR "Gabonese Republic" OR Gambia OR Gaza OR "Georgia Republic" OR "Georgian Republic" OR Ghana OR Grenada OR Guatemala OR Guinea OR Guiana OR Guyana OR Haiti OR Honduras OR India OR Maldives OR Indonesia OR Iran OR Iraq OR Jamaica OR Jordan OR Kazakhstan OR Kazakh OR Kenya OR Kiribati OR Korea OR Kosovo OR Kyrgyzstan OR Kirghizia OR "Kyrgyz Republic" OR Kirghiz OR Kirgizstan OR "Lao PDR" OR Laos OR Lebanon OR Lesotho OR Liberia OR Libya OR Macedonia OR Madagascar OR "Malagasy Republic" OR Malaysia OR "Malaya Sabah" OR Sarawak OR Malawi OR Nyasaland OR Mali OR "Marshall Islands" OR Mauritania OR Mauritius OR "Agalega Islands" OR Mexico OR Micronesia OR Moldova OR Moldovia OR Moldovian OR Mongolia OR Montenegro OR Morocco OR Mozambique OR Myanmar OR Myanma OR Burma OR Namibia OR Nepal OR Nicaragua OR Niger OR Nigeria OR Pakistan OR Paraguay OR Peru OR Philippines OR Philipines OR Phillipines OR Phillippines OR Romania OR Rumania OR Roumania OR Russia OR Russian OR Rwanda OR Ruanda OR "Saint Lucia" OR "St Lucia" OR "Saint Vincent" OR "St Vincent" OR Grenadines OR Samoa OR "Samoan Islands" OR "Sao Tome" OR Senegal OR Serbia OR Montenegro OR "Sierra Leone" OR "Sri Lanka" OR Ceylon OR "Solomon Islands" OR Somalia OR "South Africa" OR Sudan OR Suriname OR Surinam OR Swaziland OR Syria OR Tajikistan OR Tadzhikistan OR Tadjikistan OR Tadzhik OR Tanzania OR Thailand OR Togo OR "Togolese Republic" OR Tonga OR Tunisia OR Turkey OR Turkmenistan OR Turkmen OR Uganda OR Ukraine OR USSR OR "Soviet Union" OR "Union of Soviet Socialist Republics" OR Uzbekistan OR Uzbek OR Vanuatu OR "New Hebrides" OR Venezuela OR Vietnam OR "Viet Nam" OR "West Bank" OR Yemen OR Zambia OR Zimbabwe OR Rhodesia |
| Context MeSH | "poverty"[MeSH] OR "developing countries"[MeSH] OR "medical indigency"[MeSH] OR "Africa"[MeSH] OR "Asia"[MeSH] OR "Caribbean region"[MeSH] OR "South America"[MeSH] OR "Latin America"[MeSH] OR "Central America"[MeSH] OR "vulnerable populations"[MeSH] |
| combination A | #1 OR #2 |
| combination B | #3 OR #4 |
| combination C | #5 OR #6 |
| combination D | #7 OR #8 OR #9 |
| combination E | #10 AND #11 AND #12 AND #13 |
| Date filter | 2010 - present |

Search date: 28.9.21

Hits: 383

1. PsycINFO via OvidSP

| **PCC** | **Keywords and Subject Terms** |
| --- | --- |
| Population | "Aid Recipient*" OR "Asylum Seek*" OR Beneficiar* OR "conflict affected person*" OR "conflict affected population*" OR "disaster victim*" OR "displaced population*" OR "displaced people*" OR "displaced person*" Or "internally displaced" OR "internally displaced population*" OR "internally displaced people*" OR "internally displaced person*" OR evacuat* OR "forcibly displace*" OR "forcibly displaced population*" OR "forcibly displaced people*" OR "forcibly displaced person*" OR "political asylum seek*" OR "political refugee*" OR refugee* OR rescue* OR victim* |
| Population Subject terms | exp Refugees/ |
| Concept 1 | computer* OR "computer-based" OR "cyber medicine*" OR "digital electronic*" OR "digital technolog*" OR ehealth OR e-health OR "e health" OR "expert system*" OR "health 2.0" OR "mobile health" OR internet OR mhealth OR m-health OR "m health" OR mobile* OR "mobile technologies*" OR "digital health" OR SMS OR "text message*" OR telehealth OR telemedicine OR "web health" OR "smart phone*" OR laptop* OR tablet* OR app OR apps |
| Concept 1 Subject terms | exp "Digital Technology"/ OR "exp Expert Systems"/ OR exp "Telemedicine"/ OR exp "Smartphones"/ OR exp "Mobile applications"/ |
| Concept 2 | accountabilit* OR "social accountabilit*" OR "community action*" OR "patient activation*" OR adapt* OR communitarianism OR "community action*" OR "community involvement*" OR "consumer involvement*" OR "consumer participation*" OR "patient empowerment*" OR empowerment* OR engagement* OR "patient engagement*" OR "stakeholder engagement*" OR feedback* OR individualis* OR individualiz* OR "community involvement*" OR "user engagement*" OR "social obligation*" OR "obligations societ*" OR "patient participation rate*" OR "community participation" OR "patient participation" OR "patient activation" OR "patient participation rate"* OR personalis* OR personaliz* OR "public participation" OR "social responsibilit*" OR "social accountabilit*" OR "social behaviour*" OR "social obligation*" OR localisation OR localised OR localized OR localization OR "stakeholder engagement*" OR "stakeholder role*" OR tailor* OR "patient participation" OR "participatory appraisal*" OR "emancipatory intervention*" OR empowerment* OR "collaborative inquir*" OR "collaborative enquir*" OR "social reconnaissance" OR "community-based participatory intervention*" OR "community-based participatory action*" OR "user-centered" OR "user-designed" OR "user-led" OR "user centred" OR "user designed" OR "user led" OR "user-centered" OR "user centered" OR "accountability affected people" OR "accountability affected person*" OR "accountability affected populations" OR "beneficiary engagement" OR "person-centred" OR "person centred" OR "person-centered" OR "person centered" OR "social behavior*" |
| Concept 2 Subject terms | exp "Social Responsibility"/ OR exp "Community Involvement"/ OR exp "Client Participation"/ OR exp "Social Behavior"/ OR exp Empowerment/ |
| Context | "absolute povert*" OR "developing countr*" OR "extreme povert*" OR indigenc* OR indigent* OR LIC OR LMIC OR "low GDP" OR "low GNP" OR "low gross domestic product" OR "low gross national product" OR "low resource*" OR "low income" OR "low income population*" OR "low resource population*" OR MIC OR "middle income*" OR "middle income countr*" OR "middle resource*" OR poverty* OR "third world" OR "3rd world" OR "transitional context*" OR "transitional nation*" OR "transitional countr*" OR "transitional state*" OR "under-developed" OR "under-developing" OR "under developed" OR "under developing" OR underdeveloped OR "low income econom*" OR "low middle income econom*" OR Africa OR Asia OR Caribbean OR "West Indies" OR "South America" OR "Latin America" OR "Central America" OR underserved OR "under served" OR under-served OR poor OR deprived OR colonised OR colonized |
| Cochrane LMIC filter | Afghanistan OR Albania OR Algeria OR Angola OR Armenia OR Armenian OR Azerbaijan OR Bangladesh OR Benin OR Byelarus OR Byelorussian OR Belarus OR Belorussian OR Belorussia OR Belize OR Bhutan OR Bolivia OR Bosnia OR Herzegovina OR Hercegovina OR Botswana OR Brasil OR Brazil OR Bulgaria OR "Burkina Faso" OR "Burkina Fasso" OR Burundi OR Urundi OR Cambodia OR "Khmer Republic" OR Cameroon OR Cameroons OR Cameron OR Camerons OR "Cape Verde" OR "Central African Republic" OR Chad OR China OR Colombia OR Comoros OR "Comoro Islands" OR Comores OR Congo OR Zaire OR "Costa Rica" OR "Cote d’Ivoire" OR "Ivory Coast" OR Cuba OR Djibouti OR Dominica OR "Dominican Republic" OR "East Timor" OR "East Timur" OR "Timor Leste" OR Ecuador OR Egypt OR "United Arab Republic" OR "El Salvador" OR Eritrea OR Ethiopia OR Fiji OR Gabon OR "Gabonese Republic" OR Gambia OR Gaza OR "Georgia Republic" OR "Georgian Republic" OR Ghana OR Grenada OR Guatemala OR Guinea OR Guiana OR Guyana OR Haiti OR Honduras OR India OR Maldives OR Indonesia OR Iran OR Iraq OR Jamaica OR Jordan OR Kazakhstan OR Kazakh OR Kenya OR Kiribati OR Korea OR Kosovo OR Kyrgyzstan OR Kirghizia OR "Kyrgyz Republic" OR Kirghiz OR Kirgizstan OR "Lao PDR" OR Laos OR Lebanon OR Lesotho OR Liberia OR Libya OR Macedonia OR Madagascar OR "Malagasy Republic" OR Malaysia OR "Malaya Sabah" OR Sarawak OR Malawi OR Nyasaland OR Mali OR "Marshall Islands" OR Mauritania OR Mauritius OR "Agalega Islands" OR Mexico OR Micronesia OR Moldova OR Moldovia OR Moldovian OR Mongolia OR Montenegro OR Morocco OR Mozambique OR Myanmar OR Myanma OR Burma OR Namibia OR Nepal OR Nicaragua OR Niger OR Nigeria OR Pakistan OR Paraguay OR Peru OR Philippines OR Philipines OR Phillipines OR Phillippines OR Romania OR Rumania OR Roumania OR Russia OR Russian OR Rwanda OR Ruanda OR "Saint Lucia" OR "St Lucia" OR "Saint Vincent" OR "St Vincent" OR Grenadines OR Samoa OR "Samoan Islands" OR "Sao Tome" OR Senegal OR Serbia OR Montenegro OR "Sierra Leone" OR "Sri Lanka" OR Ceylon OR "Solomon Islands" OR Somalia OR "South Africa" OR Sudan OR Suriname OR Surinam OR Swaziland OR Syria OR Tajikistan OR Tadzhikistan OR Tadjikistan OR Tadzhik OR Tanzania OR Thailand OR Togo OR "Togolese Republic" OR Tonga OR Tunisia OR Turkey OR Turkmenistan OR Turkmen OR Uganda OR Ukraine OR USSR OR "Soviet Union" OR "Union of Soviet Socialist Republics" OR Uzbekistan OR Uzbek OR Vanuatu OR "New Hebrides" OR Venezuela OR Vietnam OR "Viet Nam" OR "West Bank" OR Yemen OR Zambia OR Zimbabwe OR Rhodesia |
| Context Subject terms | exp "Poverty"/ OR exp "Developing Countries"/ OR exp "At Risk Populations"/ |
| combination A | 1 OR 2 |
| combination B | 3 OR 4 |
| combination C | 5 OR 6 |
| combination D | 7 OR 8 OR 9 |
| combination E | 10 AND 11 AND 12 AND 13 |
| Date filter | 2010 - present |

Search date: 21.9.21

Hits: 308

1. Sociological abstracts via ProQuest

| **PCC** | **Keywords and Subject Terms** |
| --- | --- |
| Population | NOFT("Aid Recipient*" OR "Asylum Seek*" OR Beneficiar* OR "conflict affected person*" OR "conflict affected population*" OR "disaster victim*" OR "displaced population*" OR "displaced people*" OR "displaced person*" Or "internally displaced" OR "internally displaced population*" OR "internally displaced people*" OR "internally displaced person*" OR evacuat* OR "forcibly displace*" OR "forcibly displaced population*" OR "forcibly displaced people*" OR "forcibly displaced person*" OR "political asylum seek*" OR "political refugee*" OR refugee* OR rescue* OR victim*) |
| Population Subject terms | su.explode(Refugees); |
| Concept 1 | NOFT(computer* OR "computer-based" OR "cyber medicine*" OR "digital electronic*" OR "digital technolog*" OR ehealth OR e-health OR "e health" OR "expert system*" OR "health 2.0" OR "mobile health" OR internet OR mhealth OR m-health OR "m health" OR mobile* OR "mobile technologies*" OR "digital health" OR SMS OR "text message*" OR telehealth OR telemedicine OR "web health" OR "smart phone*" OR laptop* OR tablet* OR app OR apps) |
| Concept 1 Subject terms | su.explode("Expert Systems"); |
| Concept 2 | NOFT(accountabilit* OR "social accountabilit*" OR "community action*" OR "patient activation*" OR adapt* OR communitarianism OR "community action*" OR "community involvement*" OR "consumer involvement*" OR "consumer participation*" OR "patient empowerment*" OR empowerment* OR engagement* OR "stakeholder engagement*" OR feedback* OR individualis* OR individualiz* OR "community involvement*" OR "user engagement*" OR "patient engagement*" OR "social obligation*" OR "obligations societ*" OR "patient participation rate*" OR "community participation" OR "patient participation" OR "patient activation" OR "patient participation rate*" OR personalis* OR personaliz* OR "public participation" OR "social responsibilit*" OR "social accountabilit*" OR "social behaviour*" OR "social obligation*" OR localisation OR localised OR localized OR localization OR "stakeholder engagement*" OR "stakeholder role*" OR tailor* OR "patient participation" OR "participatory appraisal*" OR "emancipatory intervention*" OR empowerment* OR "collaborative inquir*" OR "collaborative enquir*" OR "social reconnaissance" OR "community-based participatory intervention*" OR "community-based participatory action*" OR "user-centered" OR "user-designed" OR "user-led" OR "user centred" OR "user designed" OR "user led" OR "user-centered" OR "user centered" OR "accountability affected people" OR "accountability affected person*" OR "accountability affected populations" OR "beneficiary engagement" OR "person-centred" OR "person centred" OR "person-centered" OR "person centered" OR "social behavior*") |
| Concept 2 Subject terms | su.explode("Social Responsibility"); OR su.explode("Community Involvement"); OR su.explode("Client Satisfaction"); OR su.explode("Social Behavior"); OR su.explode("Empowerment"); |
| Context | NOFT("absolute povert*" OR "developing countr*" OR "extreme povert*" OR indigenc* OR indigent* OR LIC OR LMIC OR "low GDP" OR "low GNP" OR "low gross domestic product" OR "low gross national product" OR "low resource*" OR "low income" OR "low income population*" OR "low resource population*" OR MIC OR "middle income*" OR "middle income countr*" OR "middle resource*" OR poverty* OR "third world" OR "3rd world" OR "transitional context*" OR "transitional nation*" OR "transitional countr*" OR "transitional state*" OR "under-developed" OR "under-developing" OR "under developed" OR "under developing" OR underdeveloped OR "low income econom*" OR "low middle income econom*" OR Africa OR Asia OR Caribbean OR "West Indies" OR "South America" OR "Latin America" OR "Central America" OR underserved OR "under served" OR under-served OR poor OR deprived OR colonised OR colonized) |
| Cochrane LMIC filter | NOFT(Afghanistan OR Albania OR Algeria OR Angola OR Armenia OR Armenian OR Azerbaijan OR Bangladesh OR Benin OR Byelarus OR Byelorussian OR Belarus OR Belorussian OR Belorussia OR Belize OR Bhutan OR Bolivia OR Bosnia OR Herzegovina OR Hercegovina OR Botswana OR Brasil OR Brazil OR Bulgaria OR "Burkina Faso" OR "Burkina Fasso" OR Burundi OR Urundi OR Cambodia OR "Khmer Republic" OR Cameroon OR Cameroons OR Cameron OR Camerons OR "Cape Verde" OR "Central African Republic" OR Chad OR China OR Colombia OR Comoros OR "Comoro Islands" OR Comores OR Congo OR Zaire OR "Costa Rica" OR "Cote d’Ivoire" OR "Ivory Coast" OR Cuba OR Djibouti OR Dominica OR "Dominican Republic" OR "East Timor" OR "East Timur" OR "Timor Leste" OR Ecuador OR Egypt OR "United Arab Republic" OR "El Salvador" OR Eritrea OR Ethiopia OR Fiji OR Gabon OR "Gabonese Republic" OR Gambia OR Gaza OR "Georgia Republic" OR "Georgian Republic" OR Ghana OR Grenada OR Guatemala OR Guinea OR Guiana OR Guyana OR Haiti OR Honduras OR India OR Maldives OR Indonesia OR Iran OR Iraq OR Jamaica OR Jordan OR Kazakhstan OR Kazakh OR Kenya OR Kiribati OR Korea OR Kosovo OR Kyrgyzstan OR Kirghizia OR "Kyrgyz Republic" OR Kirghiz OR Kirgizstan OR "Lao PDR" OR Laos OR Lebanon OR Lesotho OR Liberia OR Libya OR Macedonia OR Madagascar OR "Malagasy Republic" OR Malaysia OR "Malaya Sabah" OR Sarawak OR Malawi OR Nyasaland OR Mali OR "Marshall Islands" OR Mauritania OR Mauritius OR "Agalega Islands" OR Mexico OR Micronesia OR Moldova OR Moldovia OR Moldovian OR Mongolia OR Montenegro OR Morocco OR Mozambique OR Myanmar OR Myanma OR Burma OR Namibia OR Nepal OR Nicaragua OR Niger OR Nigeria OR Pakistan OR Paraguay OR Peru OR Philippines OR Philipines OR Phillipines OR Phillippines OR Romania OR Rumania OR Roumania OR Russia OR Russian OR Rwanda OR Ruanda OR "Saint Lucia" OR "St Lucia" OR "Saint Vincent" OR "St Vincent" OR Grenadines OR Samoa OR "Samoan Islands" OR "Sao Tome" OR Senegal OR Serbia OR Montenegro OR "Sierra Leone" OR "Sri Lanka" OR Ceylon OR "Solomon Islands" OR Somalia OR "South Africa" OR Sudan OR Suriname OR Surinam OR Swaziland OR Syria OR Tajikistan OR Tadzhikistan OR Tadjikistan OR Tadzhik OR Tanzania OR Thailand OR Togo OR "Togolese Republic" OR Tonga OR Tunisia OR Turkey OR Turkmenistan OR Turkmen OR Uganda OR Ukraine OR USSR OR "Soviet Union" OR "Union of Soviet Socialist Republics" OR Uzbekistan OR Uzbek OR Vanuatu OR "New Hebrides" OR Venezuela OR Vietnam OR "Viet Nam" OR "West Bank" OR Yemen OR Zambia OR Zimbabwe OR Rhodesia) |
| Context  Subject terms | su.explode("Poverty"); OR su.explode("Developing Countries"); |
| combination A | 1 OR 2 |
| combination B | 3 OR 4 |
| combination C | 5 OR 6 |
| combination D | 7 OR 8 OR 9 |
| combination E | 10 AND 11 AND 12 AND 13 |
| Date filter | 2010 - present |

Search date: 21.9.21

Hits: 117

1. Web of Science (Classic Portal) via Clarivate Analytics

| **PCC** | **Keywords** |
| --- | --- |
| Population | TS=("Aid Recipient*" OR "Asylum Seek*" OR Beneficiar* OR "conflict affected person*" OR "conflict affected population*" OR "disaster victim*" OR "displaced population*" OR "displaced people*" OR "displaced person*" Or "internally displaced" OR "internally displaced population*" OR "internally displaced people*" OR "internally displaced person*" OR evacuat* OR "forcibly displace*" OR "forcibly displaced population*" OR "forcibly displaced people*" OR "forcibly displaced person*" OR "political asylum seek*" OR "political refugee*" OR refugee* OR rescue* OR victim*) |
| Concept 1 | TS=(computer* OR "computer-based" OR "cyber medicine*" OR "digital electronic*" OR "digital technolog*" OR ehealth OR e-health OR "e health" OR "expert system*" OR "health 2.0" OR "mobile health" OR internet OR mhealth OR m-health OR "m health" OR mobile* OR "mobile technologies*" OR "digital health" OR SMS OR "text message*" OR telehealth OR telemedicine OR "web health" OR "smart phone*" OR laptop* OR tablet* OR app OR apps) |
| Concept 2 | TS=(accountabilit* OR "social accountabilit*" OR "community action*" OR "patient activation*" OR adapt* OR communitarianism OR "community action*" OR "community involvement*" OR "consumer involvement*" OR "consumer participation*" OR "patient empowerment*" OR empowerment* OR engagement* OR "patient engagement*" OR "stakeholder engagement*" OR feedback* OR individualis* OR individualiz* OR "community involvement*" OR "user engagement*" OR "social obligation*" OR "obligations societ*" OR "patient participation rate*" OR "community participation" OR "patient activation" OR "patient participation rate"* OR personalis* OR personaliz* OR "public participation" OR "social responsibilit*" OR "social accountabilit*" OR "social behaviour*" OR "social obligation*" OR "social behavior*" OR localisation OR localised OR localized OR localization OR "stakeholder engagement*" OR "stakeholder role*" OR tailor* OR "patient participation" OR "participatory appraisal*" OR "emancipatory intervention*" OR empowerment* OR "collaborative inquir*" OR "collaborative enquir*" OR "social reconnaissance" OR "community-based participatory intervention*" OR "community-based participatory action*" OR "user-centered" OR "user-designed" OR "user-led" OR "user centred" OR "user designed" OR "user led" OR "user-centered" OR "user centered" OR "accountability affected people" OR "accountability affected person*" OR "accountability affected populations" OR "beneficiary engagement" OR "person-centred" OR "person centred" OR "person-centered" OR "person centered") |
| Context | TS=("absolute povert*" OR "developing countr*" OR "extreme povert*" OR indigenc* OR indigent* OR LIC OR LMIC OR "low GDP" OR "low GNP" OR "low gross domestic product" OR "low gross national product" OR "low resource*" OR "low income" OR "low income population*" OR "low resource population*" OR MIC OR "middle income*" OR "middle income countr*" OR "middle resource*" OR poverty* OR "third world" OR "3rd world" OR "transitional context*" OR "transitional nation*" OR "transitional countr*" OR "transitional state*" OR "under-developed" OR "under-developing" OR "under developed" OR "under developing" OR underdeveloped OR "low income econom*" OR "low middle income econom*" OR Africa OR Asia OR Caribbean OR "West Indies" OR "South America" OR "Latin America" OR "Central America" OR underserved OR "under served" OR under-served OR poor OR deprived OR colonised OR colonized) |
| Cochrane LMIC filter | TS=(Afghanistan OR Albania OR Algeria OR Angola OR Armenia OR Armenian OR Azerbaijan OR Bangladesh OR Benin OR Byelarus OR Byelorussian OR Belarus OR Belorussian OR Belorussia OR Belize OR Bhutan OR Bolivia OR Bosnia OR Herzegovina OR Hercegovina OR Botswana OR Brasil OR Brazil OR Bulgaria OR "Burkina Faso" OR "Burkina Fasso" OR Burundi OR Urundi OR Cambodia OR "Khmer Republic" OR Cameroon OR Cameroons OR Cameron OR Camerons OR "Cape Verde" OR "Central African Republic" OR Chad OR China OR Colombia OR Comoros OR "Comoro Islands" OR Comores OR Congo OR Zaire OR "Costa Rica" OR "Cote d’Ivoire" OR "Ivory Coast" OR Cuba OR Djibouti OR Dominica OR "Dominican Republic" OR "East Timor" OR "East Timur" OR "Timor Leste" OR Ecuador OR Egypt OR "United Arab Republic" OR "El Salvador" OR Eritrea OR Ethiopia OR Fiji OR Gabon OR "Gabonese Republic" OR Gambia OR Gaza OR "Georgia Republic" OR "Georgian Republic" OR Ghana OR Grenada OR Guatemala OR Guinea OR Guiana OR Guyana OR Haiti OR Honduras OR India OR Maldives OR Indonesia OR Iran OR Iraq OR Jamaica OR Jordan OR Kazakhstan OR Kazakh OR Kenya OR Kiribati OR Korea OR Kosovo OR Kyrgyzstan OR Kirghizia OR "Kyrgyz Republic" OR Kirghiz OR Kirgizstan OR "Lao PDR" OR Laos OR Lebanon OR Lesotho OR Liberia OR Libya OR Macedonia OR Madagascar OR "Malagasy Republic" OR Malaysia OR "Malaya Sabah" OR Sarawak OR Malawi OR Nyasaland OR Mali OR "Marshall Islands" OR Mauritania OR Mauritius OR "Agalega Islands" OR Mexico OR Micronesia OR Moldova OR Moldovia OR Moldovian OR Mongolia OR Montenegro OR Morocco OR Mozambique OR Myanmar OR Myanma OR Burma OR Namibia OR Nepal OR Nicaragua OR Niger OR Nigeria OR Pakistan OR Paraguay OR Peru OR Philippines OR Philipines OR Phillipines OR Phillippines OR Romania OR Rumania OR Roumania OR Russia OR Russian OR Rwanda OR Ruanda OR "Saint Lucia" OR "St Lucia" OR "Saint Vincent" OR "St Vincent" OR Grenadines OR Samoa OR "Samoan Islands" OR "Sao Tome" OR Senegal OR Serbia OR Montenegro OR "Sierra Leone" OR "Sri Lanka" OR Ceylon OR "Solomon Islands" OR Somalia OR "South Africa" OR Sudan OR Suriname OR Surinam OR Swaziland OR Syria OR Tajikistan OR Tadzhikistan OR Tadjikistan OR Tadzhik OR Tanzania OR Thailand OR Togo OR "Togolese Republic" OR Tonga OR Tunisia OR Turkey OR Turkmenistan OR Turkmen OR Uganda OR Ukraine OR USSR OR "Soviet Union" OR "Union of Soviet Socialist Republics" OR Uzbekistan OR Uzbek OR Vanuatu OR "New Hebrides" OR Venezuela OR Vietnam OR "Viet Nam" OR "West Bank" OR Yemen OR Zambia OR Zimbabwe OR Rhodesia) |
| combination A | #4 OR #5 |
| combination B | #1 AND #2 AND #3 AND #6 |
| Date filter | 2010 - present |

Search date: 21.9.21

Hits: 223

1. International Committee of the Red Cross’s resource portal (ICRC)

| **Name** | **Search Terms + 2010> filter** |
| --- | --- |
| ICRC | digital AND health AND local |

Search date: 9.11.2021

Hits: 25

1. Oxfam’s Open Library Repository (Oxfam)

| **Name** | **Search Terms + 2010> filter** |
| --- | --- |
| Oxfam | digital AND health AND local |

Search date: 29.9.2021

Hits: 164

1. Active Learning Network for Accountability and Performance’s resource portal (ALNAP

| **Name** | **Search Terms + 2010> filter** |
| --- | --- |
| ALNAP | digital AND health AND local |

Search dates: 29.9.2021

Hits: 1917

_________________________________________________________________________________

**Hits Summary:**

|  | Source | # Hits Total | Date Entered into EndNote20 | Date Entered into Covidence | Duplicates found in Covidence | Totals hits in Covidence after de-duplication |
| --- | --- | --- | --- | --- | --- | --- |
| 1 | CINAHL via EBSCO | 62 | 28.9.2021 | 28.9.2021 | 14 | 48 |
| 2 | Medline via PubMed | 383 | 28.9.2021 | 28.9.2021 | 0 | 383 |
| 3 | PsycINFO via OvidSP | 308 | 28.9.2021 | 28.9.2021 | 68 | 240 |
| 4 | Sociological Abstracts via ProQuest | 117 | 28.9.2021 | 28.9.2021 | 8 | 109 |
| 5 | Web of Science Classic via Clarivate Analytics | 223 | 28.9.2021 | 28.9.2021 | 32 | 191 |
| 6 | ICRC | 25 | 9.11.2021 | 9.11.2021 | 0 | 25 |
| 7 | Oxfam | 164 | 29.9.2021 | 9.11.2021 | 4 | 160 |
| 8 | ALNAP | 1917 | 29.9.2021 – 9.11.2021 | 9.11.2021 | 16 | 1901 |
|  |  | **3,199** |  |  | 142 | **3,057** |
