# Supplementary material for: Localisation of digital health tools used by displaced populations in low and middle-income settings: a scoping review and critical analysis of the Participation Revolution
Source: Confl Health. 2023 Apr 15;17:20. doi: 10.1186/s13031-023-00518-9 (PMC10105546; doi:10.1186/s13031-023-00518-9)
Supplement: Supplementary file 2 — Additional file 2. PRISMA-ScR flow chart. [file 13031_2023_518_MOESM2_ESM.docx]

**Identification of studies via databases and registers**

Records removed before screening:

Duplicate records removed (n = 142)

Records identified (n= 3199) from:

5 Databases (n = 1093)

3 Registers (n = 2106)

**Identification**

Records (title and abstract) screened

(n = 3057)

Records excluded due to exclusion criteria

(n = 2965)

Reports sought for retrieval

(n = 92)

Reports not retrieved

(n = 0)

**Screening**

Reports excluded:

Reports excluded (n = 65)

Wrong intervention (n =45)

Wrong patient population (n = 11)

Wrong setting (n = 7)

Wrong outcomes (n = 1)

Wrong route of administration (n = 1)

Reports assessed (full text) for eligibility

(n = 92)

Total papers included (n=27)

Studies included in synthesis

(n = 19)

Reports included in synthesis

(n = 8)

**Included**

*From:*  Page MJ, McKenzie JE, Bossuyt PM, Boutron I, Hoffmann TC, Mulrow CD, et al. The PRISMA 2020 statement: an updated guideline for reporting systematic reviews. BMJ 2021;372:n71. doi: 10.1136/bmj.n71

For more information, visit: <http://www.prisma-statement.org/>
