# Supplementary material for: Localisation of digital health tools used by displaced populations in low and middle-income settings: a scoping review and critical analysis of the Participation Revolution
Source: Confl Health. 2023 Apr 15;17:20. doi: 10.1186/s13031-023-00518-9 (PMC10105546; doi:10.1186/s13031-023-00518-9)
Supplement: Supplementary file 3 — Additional file 3. Data chart. [file 13031_2023_518_MOESM3_ESM.docx]

| Paper Overview | | | | | | | | | Population | Context | | | Concept | Results | Localisation & Participation |
| --- | --- | --- | --- | --- | --- | --- | --- | --- | --- | --- | --- | --- | --- | --- | --- |
| Ref: | Title of paper | Author and year of publication | Authorship affiliations | Publication Type | Study design & overview | Health issue(s) addressed | Study size | Study duration | Population of interest | Humanitarian context | Economic classification of context | Displacement status & context | Digital health tool | Key Findings | Involvement of local groups or displaced people |
| (1) | mHealth for mental health in the Middle East: Need, technology use, and readiness among Palestinians in the West Bank | Dror Ben-Zeev et al. 2017 | USA; University | Peer reviewed academic Journal | Cross- sectional study: survey, systematic evaluation of readiness towards mobile phone use for mental health | Mental health | 272 | Not stated | Palestinian adults in urban, rural, and refugee camp settings | West Bank, Palestine | Lower middle income | Refugee; conflict; Internal displacement | None | High smartphone ownership and social media usage, without different gender access. Connectivity was reported to be widespread and generally reliable | Participation by completion of surveys, using Palestinian team. Recognises interventions should be informed by cultural, historical, and geopolitical context |
| (2) | Sijilli: a mobile electronic health records system for refugees in low-resource settings | Shadi Saleh et al. 2019 | Lebanon; University | Peer reviewed academic Journal | Case report of Sijilli system as mobile records system to support for at-risk populations | Allergies, surgeries, medication use & depression screening | > 8000 | N/A | Refugee populations | Lebanon | Middle income/ resource | Refugee; conflict | “Sijilli”, electronic health records (EHR) for general health management | Advocates for cloud-based electronic health records. No health outcomes assessed | No involvement stated |
| (3) | mHealth use for non-communicable disease (NCD) care in primary health: patients' perspective from rural settings and refugee camps | Shadi Saleh et al. 2018 | Lebanon, Canada, UAE; University | Peer reviewed academic Journal | Mixed-methods design, case control study evaluating the eSahha SMS community screening in rural and refugee camps | NCDs: hypertension and diabetes | 1000 | 1-year intervention period | Rural refugee groups | Lebanon | Middle income/ resource | Refugee (rural) | “eSahha” short-messaging system (SMS) for NCD management | High perception of SMS as useful and easy to understand. Med-high self-reported daily behaviour modification compliance but lower among women, older age groups, unemployed and illiterate individuals and those with only elementary education | Focus group discussions and phone surveys informed intervention |
| (4) | Did Bangladesh miss the opportunity to use telepsychiatry in the Rohingya refugee crisis? | Tanjir Rashid Soron at el. 2019 | Bangladesh, India; Business | Peer reviewed academic Journal | Correspondence article on the use of telepsychiatry with refugees | mental health, mental disorders | N/A | N/A | Refugee populations | Bangladesh | Middle income/ resource | Refugee; conflict | No specific digital health promotion approaches discussed | No health outcomes assessed | No involvement stated |
| (5) | Integrating Health technologies in Health Services for Syrian Refugees in Lebanon: A Qualitative study | Reem Talhouk at el. 2020 | UK, Lebanon, Sweden, Australia; University | Peer reviewed academic Journal | Qualitative study understanding the potential for technology integration in Lebanon's primary health care | General health | 17 | Data collection: 3 months | Syrian refugees | Lebanon | Middle income/ resource | Refugee; conflict | No specific digital health promotion approaches discussed | Lack of resources and time alongside varying health and technology literacy were the main challenges for integrating technologies within health care services. Refugees were viewed as a mobile population thus health care centres were less willing to invest | Semi-structured interviews from health providers and key informants, using language around empowerment |
| (6) | Use of short messaging services to assess depressive symptoms among refugees in South Africa: Implications for social services providing mental health care in resource-poor settings | Andrew Tomita at el. 2016 | South Africa & USA; University | Peer reviewed academic Journal | Prospective cohort study feasibility of SMS-based methods to screen for depression risk and compare its reliability and acceptability with face-to-face consultation | Depressive symptoms, mental health | 153 | Data collection: 10 months | Refugee populations | South Africa | Middle income/ resource | Refugee | Unnamed short-messaging system (SMS) screening for depression | There was a fair reliability between face-to-face and SMS-based methods with no significant preference rating between the two methods | Refugees participated in screening assessments. Recognition of culture, tone and language as factors impacting user interactions with digital tools |
| (7) | Utilization of Mobile Mental Health Services among Syrian Refugees and Other Vulnerable Arab Populations-A Systematic Review | Adeel Ashfaq at el. 2020 | USA; Private health organisation | Peer reviewed Academic Journal | Systematic literature review evaluating mMHealth uptake, acceptability, feasibility, barriers and adaption to cultural and gender norms | Mental health, post-traumatic stress disorder, psychological stress | 10 papers | 9 Months | Participants from the Middle East, Arab Culture/Background or refugees receiving mental healthcare in home or host nations; healthcare workforce members, community members & all related parties involved in the provision of mental healthcare to the participants | "Arabic countries" (includes papers with study populations from/in Germany, Sweden, and Egypt; Saudi Arabia; Lebanon; West Bank; Afghanistan, Iraq, Turkey, Sri Lanka, and Sudan; Palestine; Syrians; Iraq; Turkey; Denmark) | Combination low, middle & high income | General humanitarian contexts | No specific digital health promotion approaches discussed | Medium-high positive interest towards mHealth. Medium-high mobile phone ownership and connectivity. Barriers included cultural, financial, technical, infrastructural and data privacy limitations and a lack of mental health awareness in the study populations | No participation. Recognises the importance of gender, language, and cultural adaptation of technologies in the uptake of digital tools and pays specific attention to culture and language (idioms) that construct local understandings of health, suffering, etc. Outlines the rejection of the bio-medical model in addressing certain health issues. Stresses the significance of stigma in mental health as a barrier to health-seeking behaviours |
| (8) | A review of telemental health in international and post-disaster settings | Eugene Augusterfer et al. 2015 | USA; University- affiliated program | Peer reviewed Academic Journal | Non-systematic literature review of mental health impact of the Haiti 2010 earthquake and limited use of telemedicine | Mental health | N/A | Not stated | Disaster affected in international and post-disaster settings | Haiti | Lower middle income | Internal displacement; disaster | Unnamed telemental health and mhealth patient vaccination records | Advocates for use of telemedicine. No health outcomes were assessed | No participation. Recognises inappropriate language and cultural expressions of suffering can lead to failure in positive health outcomes |
| (9) | eHealth for service delivery in conflict: a narrative review of the application of eHealth technologies in contemporary conflict settings | Gemma Bowsher et al. 2021 | UK, Lebanon; University | Peer reviewed academic Journal | Hermeneutic narrative literature review categorising eHealth usage in conflict | Mental health, some mention of NCDs | 46 papers | Not stated | Conflict-affected populations, defined as a population experiencing the health consequences of conflict either within a current conflict region during ongoing conflict or insurgency, or as a displaced person from an ongoing conflict. | Somalia, Sudan, Afghanistan, Syria, Iraq, Pakistan, Chechnya, Gaza and the Democratic Republic of Congo | Combination low & middle income | Refugee; conflict; internal displacement | No specific digital health promotion approaches discussed in detail | Reviewed 46 ehealth usage papers & categorised them as (1) eHealth for clinical management, (2) e-learning for healthcare in conflict and (3) eHealth for information management in conflict | No participation. Recognises ethical and cultural concerns arising from the use of digital health tools for marginalised communities |
| (10) | Children Immunization App (CImA), Low-Cost Digital Solution for Supporting Syrian Refugees in Zaatari Camp in Jordan - General Description | Ziad El-Khatib et al. 2020 | Sweden, Jordan; University | Peer reviewed academic Journal | Description study digital vaccination promotion app emphasising | Vaccinable diseases | Not stated | Not stated | Syrian refugee parents and caregivers of children at vaccine-age living in Zaatari Camp, Jordan, | Jordan | Upper middle income | Refugee | Children Immunization App “CImA”, mobile phone application with vaccination reminders for parents of new-born babies | Pilot study without published outcomes | Collaboration with local health actors but does not detail inclusion or not of community groups, message development or testing etc. |
| (11) | Use of telepsychiatry in areas of conflict: the Syrian refugee crisis as an example | Hussam Jefee-Bahloul et al. 2014 | USA; University | Peer reviewed academic Journal | Description study on the provision of mental health telepsychiatry through a humanitarian organization | Mental health | 6 | unknown | Syrian psychiatric patients living in Jordanian refugee camps identified as treatment-resistant psychiatric cases within previous treatment programmes | Jordan | Upper middle income | Refugee; conflict | Unnamed telepsychiatry mental health service | Treatment-resistant cases were reported as completed with diagnoses. Consultation from patient-treatment perspective were not measured | Involves local practitioner and recognises cultural and linguistic elements when working with patients is important |
| (12) | Agile Application of Digital Health Interventions during the COVID-19 Refugee Response | Nirmala P. Narla et al. 2020 | USA, Turkey; Private health organisation | Peer reviewed academic Journal | Evaluation/report of a feasibility pilot study of an mhealth platform to be effectively adapted populations | Maternal and child health; Covid-19 | 200 | Not stated | Women, young mothers or, pregnant or had at least one child under the age of two | Turkey | Middle income/ resource | Refugees; Covid-19 pandemic | Health Records App “HERA”, electronic health records, mobile phone application with maternal and child health services | An existing application was rapidly adapted for the purposes of symptomatic Covid-19 contacts. 75% of users were successfully contacted for symptomatic assessment at two-week intervals | Participation by intervention uptake and feedback provision. Acknowledges innovative technologies could empower displaced populations by increasing control over their own health |
| (13) | Dissemination and implementation of the e-MCH Handbook, UNRWA's newly released maternal and child health mobile application: a cross-sectional study | Seif Nasir et al. 2020 | USA, Jordan, Japan; University | Peer reviewed academic Journal | Cross-sectional study of factors associated with the dissemination and implementation of m-Health | Maternal and child health | 1042 | One week | Palestinian pregnant women or mothers of children aged 0 to 5 years who could read and write in Arabic. | Jordan | Middle income/ resource | Refugees; conflict | “e-MCH” electronic Maternal and Child Handbook, mobile phone application providing information on maternal and child health care | High mobile phone ownership, the majority being smartphones, however, at the end of the intervention, only 23.8% had downloaded the tool and only 17.4% had used it | Participation through intervention use. Study states “"No participants were involved in the development of the research questions or outcome measures or the design and conduct of this study". They recognise that sociocultural beliefs and gender roles should be examined in future digital health interventions |
| (14) | Feasibility and acceptability of mobile phone platforms to deliver interventions to address gender-based violence among Syrian adolescent girls and young women in Izmir, Turkey | Ekua Yankah et al. 2019 | France, Australia, USA, UK, Turkey; Non- governmental organisation | Peer reviewed academic Journal | Cross-sectional study user feasibility and acceptability of mobile phone platforms to address gender-based violence | gender-based violence | 29 | Not stated | Syrian refugees in Izmir, Turkey, Female adolescents; Male adolescents; Men; Women | Turkey | Middle income/ resource | Refugee; conflict | “Twilio” mobile phone application with targeted public safety and information alerts preventing GBV | High mobile phone ownership, however, gendered expectations and sharing influenced use. Key concerns included the security of personal data and data privacy. The Twillo pilot study was not undertaken | Participation through focus group discussions. Calls for future research to focus on content development through collaboration with intended beneficiaries and their families to contextualise interventions. Recognise potential biases in their work |
| (15) | User-Centered App Adaptation of a Low-Intensity E-Mental Health Intervention for Syrian Refugees | Sebastian Burchert et al. 2019 | Germany Switzerland, The Netherlands; University | Peer reviewed academic Journal | Rapid qualitative assessment describes the stages of a mobile mental health adaptation process | Mental health | Stage 1 = 128; Stage 2 = 36; Stage 3 = 32 | 6 months | Syrian refugees with mental health issues in Egypt | Germany, Sweden and Egypt. | Combination low & high income | Refugee; conflict | “Step-by-Step” mobile phone application for mental health (depression) with complimentary phone support service | Widespread use of digital technologies with a positive perception of health outcomes. Technical literacy, internet access, acceptability and credibility were main barriers | Participation through focus group discussions and key informant interviews. Recognises the importance of language and user-informed interventions. Mentions on-going participation as an intention of future work. |
| (16) | Step-by-step: Feasibility randomised controlled trial of a mobile-based intervention for depression among populations affected by adversity in Lebanon | Eva Heim et al. 2021 | Switzerland, Germany, Lebanon, The Netherlands; University | Peer reviewed academic Journal | Randomised control trial evaluates the feasibility and acceptability of research methods for an E-mental health intervention | Mental health | 138 | 3 months follow up | Syrian refugees (and Lebanese) living in Lebanon with depression | Lebanon | Upper middle income | Refugee; conflict | “Step-by-Step” mobile phone application for mental health (depression) with complimentary phone support service | Results demonstrated potential for statistically significant reduction of depression and anxiety at post-assessment and follow-up stage | Participation in the intervention. Recognises culturally adaptions and community participatory approaches as key to successful intervention activities |
| (17) | Perceptions Toward the Use of Digital Technology for Enhancing Family Planning Services: Focus Group Discussion with Beneficiaries and Key Informative Interview with Midwives | Hind Yousef et al. 2021 | Jordan; Non- governmental organisation | Peer reviewed academic Journal | Cross-sectional study of digital health perceptions to support and enhance access to family planning services | General health regarding family planning | 49 | 3 months data collection | Married women of reproductive age & midwives | Jordan | Middle income/resource | Refugee; conflict | No specific digital health promotion approaches discussed | Digital technology can be feasible, cost-effective, well accepted, and potentially beneficial in increasing awareness and knowledge regarding family planning (FP) methods and their side effects. eHealth literacy and concerns over content accuracy were main barriers | Participation through key informant interviews and focus group discussions. Recognise the importance of framing health information according to user needs and preferences, uses ‘beneficiaries’ language |
| (18) | Telepsychiatry for post-traumatic stress disorder: a call for action in the Syrian conflict | Malik Nassan et al. 2015 | USA, Peru; Private health organisation | Peer reviewed academic Journal | Expert opinion on the use of telepsychiatry as a temporary, cost-effective solution for the growing mental health care needs | Mental health, post-traumatic stress disorder | N/A | N/A | Not specified | Syria, Jordan, Turkey | Combination Low & Middle | Refugee; conflict | No specific digital health promotion approaches discussed | Advocates for telepsychiatry, outlining patient satisfaction, effectiveness, accuracy and cost-effectiveness | No involvement stated |
| (19) | From Digital Promise to Frontline Practice: New and Emerging Technologies in Humanitarian Practice | Leonie Arendt-Cassetta et al. 2021 | International; UN agency | Non-peer reviewed Grey literature | Expert opinion on new and emerging technologies in humanitarian practice | Covid-19, medical health care, mental health care, however this is a general paper | N/A | N/A | General - Those within humanitarian crises | General context focus | Combination - General/ unspecified | Refugee; Internal displacement; disaster affected; general humanitarian contexts | No specific digital health promotion approaches discussed | No intervention or health outcomes assessed | No involvement stated |
| (20) | Forced Migration Review: The Technology Issue | Marion Couldrey, Maurice Herson (eds.) et al. 2011 | UK; University | Non-peer reviewed Grey literature | Expert opinion on effects of changes in communication technology on displaced people and their implications | Malarial care; general health care | N/A | N/A | General - Those that are forcibly displaced | Haiti, Philippines, Rwanda, Pakistan, Sudan, Nepal, Kenya, India, Malaysia, Republic of Moldova, Costa Rica, Czech Republic | Combination low, middle & high income | Refugee; internal displacement; disaster affected; broad humanitarian contexts | Red Cross information telephone line regarding Cholera outbreaks & an unnamed short-messaging system (SMS) with advice regarding general infectious disease outbreaks | No intervention or health outcomes assessed | No involvement stated, discusses potential empowerment from the use of digital tools |
| (21) | Mobile Technology in Emergencies: Efficient cash transfer mechanisms and effective two-way communication with disaster-affected communities using mobile phone technology | David Hallow, 2012 | UK; Non- governmental organisation | Non-peer reviewed Grey literature | Case study on the opportunities and benefits of mobile technology in emergencies | Broad public health | N/A | N/A | General - Those that are affected by disaster/ emergency | Niger, Kenya, Somalia, Bangladesh, Afghanistan, Pakistan | Low and lower middle income | Refugee; Internal displacement; disaster affected; emergency affected; broad humanitarian contexts | No specific digital health promotion approaches discussed | No intervention or health outcomes assessed | No involvement stated, “The appropriate use of mobile phones can facilitate the effective flow of information and can empower new voices to participate and inform the humanitarian response” |
| (22) | International Federation of Red Cross and Red Crescent Societies, World Disasters Report 2013 - Focus on technology and the future of humanitarian action | International Federation of the Red Cross, 2013 | International; Non- governmental organisation | Non-peer reviewed Grey literature | Expert opinion on technology and its use in humanitarian action | Cholera | N/A | N/A | General - Those within humanitarian crises | Indonesia (Sumatra), Pakistan, Central African Republic, Sierra Leone, Philippines, Haiti, Japan, USA, | Combination low, middle & high income | Refugee; Internal displacement; disaster; conflict; broad humanitarian contexts | “Trilogy Emergency Relief Application (TERA)” short-messaging system (SMS) for disease prevention & “Universal app” and “Everyday First Aid” mobile phone applications from the American Red Cross providing first aid, health education and promotion | No intervention or health outcomes assessed | No involvement stated, “Disaster-affected people are not ‘victims’ but a significant force of first responders” |
| (23) | Displaced and Disconnected | Dr. Aaron Martin et al. 2019 | International; UN agency | Non-peer reviewed Grey literature | Expert opinion on ID authentication as a significant barrier to mobile connectivity | Mental health | N/A | N/A | General - Those within humanitarian crises | Afghanistan, Bangladesh, Brazil, Burundi, Cameroon, Central African Republic, Chad, Democratic Republic of Congo, Ethiopia, Jordan, Kenya, Lebanon, Mauritania, Niger, Nigeria, Rwanda, Tanzania, Turkey, Uganda, and Zambia | Combination Low & Middle | Asylum seekers; refugees; internal displacement; returned refugees; returned IDPs; stateless persons; broad humanitarian contexts | No specific digital health promotion approaches discussed | No intervention or health outcomes assessed | No involvement stated |
| (24) | Using digital health to enable ethical health research in conflict and other humanitarian settings | Eric D. Perakslis et al. 2018 | USA; Business | Peer reviewed academic Journal | Non-systematic review on existing interventions and knowledge related to ethics in digital health | general health | N/A | N/A | Not specified | Democratic Republic of Congo, Ethiopia, Jordan, Kenya, Lebanon, Mauritania, Niger, | Combination Low & Middle | Conflict; disaster; disease outbreak | Unnamed community surveillance mobile phone application for mapping Ebola virus outbreaks | Calls to fill ethical gaps in humanitarian research including consent, privacy and protection, data reliability and quality, provenance and transparency | No involvement. Recognises the benefits of user-centred design as a best practice method to consider long term consequences and specific delivery methodologies |
| (25) | Innovating mobile solutions for refugees in East Africa | Samuel Hall, 2018 | International; Business | Non-peer reviewed Grey literature | Case study on the opportunities and barriers to mobile technology and internet use in two refugee centres | general health | 1365 | Not stated | Those living in Kakuma refugee camp and Navikale refugee settlement | Nigeria, Rwanda, Tanzania, Turkey, Uganda, and Zambia | Combination Low & Middle | Refugee | No specific digital health promotion approaches discussed | High mobile device access amongst refugees was nuanced by age, gender and education level. Cost, weak network strength and charging issues were the main barriers. Community awareness of digital services for refugees is low and this is not impacted by age group or type of device ownership | Participation through key informant interviews and focus group discussions. Recognises intervention adaptation is important to address local needs |
| (26) | The use of Mobile Technology for Humanitarian Programming in Syria | Syria Independent Monitoring (SIM) Team, 2017 | UK; Government | Non-peer reviewed Grey literature | Non-systematic review & qualitative interview intervention on potentials and constraints within the use of mobile technology within humanitarian programming | General health | 58 | qualitative research: in the fourth quarter of 2016 | Conflict-affected populations, defined as a population experiencing the health consequences of conflict either within a current conflict region during ongoing conflict or insurgency, or as a displaced person from an ongoing conflict. | Syria | Low income/ resource | Conflict | No specific digital health promotion approaches discussed | Widespread smartphone and internet use but intermittent connectivity was reported. Existing connectivity coverage should be mapped to illustrate black spots | Participation through key informant interviews |
| (27) | Connecting Refugees: How Internet and Mobile Connectivity can Improve Refugee Well-Being and Transform Humanitarian Action | UNHCR., 2016 | International, UN agency | Non-peer reviewed Grey literature | Report on key challenges in refugees’ connectivity | General health | N/A | Not stated | Refugee populations | General context focus | Combination Low & Middle | Refugee; general humanitarian contexts | No specific digital health promotion approaches discussed | No intervention or health outcomes assessed | No participation. Recognises that tailoring global approaches to local contexts in order to implement country-specific strategies |

References

1. Ben-Zeev D, Fathy C, Jonathan G, Abuharb B, Brian RM, Kesbeh L, et al. mHealth for mental health in the Middle East: Need, technology use, and readiness among Palestinians in the West Bank. Asian J Psychiatr. 2017;27:1-4.

2. Saleh S, El Arnaout N, Faulkner JR, Sayegh MH. Sijilli: a mobile electronic health records system for refugees in low-resource settings. Lancet Glob Health. 2019;7(9):e1168-e9.

3. Saleh S, Farah A, El Arnaout N, Dimassi H, El Morr C, Muntaner C, et al. mHealth use for non-communicable diseases care in primary health: patients' perspective from rural settings and refugee camps. Journal of Public Health. 2018;40:52-63.

4. Soron TR, Heanoy EZ, Udayasankaran JG. Did Bangladesh miss the opportunity to use telepsychiatry in the rohingya refugee crisis? The Lancet Psychiatry. 2019;6(5):374.

5. Talhouk R, Akik C, Araujo-Soares V, Ahmad B, Mesmar S, Olivier P, et al. Integrating Health Technologies in Health Services for Syrian Refugees in Lebanon: Qualitative Study. J Med Internet Res. 2020;22(7):e14283.

6. Tomita A, Kandolo KM, Susser E, Burns JK. Use of short messaging services to assess depressive symptoms among refugees in South Africa: Implications for social services providing mental health care in resource-poor settings. J Telemed Telecare. 2016;22(6):369-77.

7. Ashfaq A, Esmaili S, Najjar M, Batool F, Mukatash T, Al-Ani HA, et al. Utilization of Mobile Mental Health Services among Syrian Refugees and Other Vulnerable Arab Populations-A Systematic Review. Int J Environ Res Public Health. 2020;17(4):15.

8. Augusterfer EF, Mollica RF, Lavelle J. A review of telemental health in international and post-disaster settings. Int Rev Psychiatry. 2015;27(6):540-6.

9. Bowsher G, El Achi N, Augustin K, Meagher K, Ekzayez A, Roberts B, et al. eHealth for service delivery in conflict: a narrative review of the application of eHealth technologies in contemporary conflict settings. Health Policy Plan. 2021;36(6):974-81.

10. El-Khatib Z, El-Halabi S, Abu Khdeir M, Khader YS. Children Immunization App (CImA), Low-Cost Digital Solution for Supporting Syrian Refugees in Zaatari Camp in Jordan - General Description. Stud Health Technol Inform. 2020;271:117-20.

11. Jefee-Bahloul H. Use of telepsychiatry in areas of conflict: the Syrian refugee crisis as an example. J Telemed Telecare. 2014;20(3):167-8.

12. Narla NP, Surmeli A, Kivlehan SM. Agile Application of Digital Health Interventions during the COVID-19 Refugee Response. Ann Glob Health. 2020;86(1):5.

13. Nasir S, Goto R, Kitamura A, Alafeef S, Ballout G, Hababeh M, et al. Dissemination and implementation of the e-MCH H andbook, UNRWA's newly released maternal and child health mobile application: a cross-sectional study. BMJ Open. 2020;10(3):e034885.

14. Yankah E, Mohamed O, Wringe A, Afaneh O, Saleh M, Speed O, et al. Feasibility and acceptability of mobile phone platforms to deliver interventions to address gender-based violence among Syrian adolescent girls and young women in Izmir, Turkey. Vulnerable Children & Youth Studies. 2020;15(2):133-43.

15. Burchert S, Alkneme MS, Bird M, Carswell K, Cuijpers P, Hansen P, et al. User-Centered App Adaptation of a Low-Intensity E-Mental Health Intervention for Syrian Refugees. Frontiers in Psychiatry. 2019;9(663).

16. Heim E, Ramia JA, Hana RA, Burchert S, Carswell K, Cornelisz I, et al. Step-by-step: Feasibility randomised controlled trial of a mobile-based intervention for depression among populations affected by adversity in Lebanon. Internet Interv. 2021;24:9.

17. Yousef H, Al-Sheyab N, Al Nsour M, Khader Y, Al Kattan M, Bardus M, et al. Perceptions Toward the Use of Digital Technology for Enhancing Family Planning Services: Focus Group Discussion With Beneficiaries and Key Informative Interview With Midwives. J Med Internet Res. 2021;23(7):e25947.

18. Nassan M, Frye MA, Adi A, Alarcon RD. Telepsychiatry for post-traumatic stress disorder: A call for action in the Syrian conflict. The Lancet Psychiatry. 2015;2(10):866.

19. Arendt-Cassetta L. From Digital Promise to Frontline Practice: New and Emerging Technologies in Humanitarian Action [Online Report]. Geneva: OCHA; 2021 [cited 2021 4 October]. Available from: <https://www.alnap.org/help-library/from-digital-promise-to-frontline-practice-new-and-emerging-technologies-in>.

20. M. C, Herson M. Forced Migration Review: Armed non-state actors and displacement [Online Report]. Oxford, UK: Refugee Studies Centre; 2011 [cited 2021 8 November]. Available from: <https://www.alnap.org/help-library/forced-migration-review-armed-non-state-actors-and-displacement>.

21. Hallow D, Mitchell J, Gladwell C, Aggiss R. Mobile Technology in Emergencies: Efficient cash transfer mechanisms and effective two-way communication with disaster-affected communities using mobile phone technology [Online Report]. UK: Save the Children; 2012 [cited 2021 3 November]. Available from: <https://www.alnap.org/help-library/mobile-technology-in-emergencies-efficient-cash-transfer-mechanisms-and-effective-two>.

22. International Federation of Red Cross and Red Crescent Societies. World Disasters Report 2013 - Focus on technology and the future of humanitarian action [Online Report]. Geneva: IFRC; 2013 [cited 2021 2 November]. Available from: <https://www.alnap.org/help-library/world-disasters-report-2013-focus-on-technology-and-the-future-of-humanitarian-action>.

23. Martin A. Displaced and Disconnected [Online Report]. Unknown: UNHCR; 2019 [cited 2021 18 October]. Available from: <https://www.alnap.org/help-library/displaced-and-disconnected-0>.

24. Perakslis ED. Using digital health to enable ethical health research in conflict and other humanitarian settings. Conflict and Health. 2018;12(1):23.

25. Samuel Hall. Innovating mobile solutions for refugees in East Africa [Online Report]. Unknown: Samuel Hall; 2021 [cited 2021 20 October]. Available from: <https://www.alnap.org/help-library/innovating-mobile-solutions-for-refugees-in-east-africa>.

26. Syria Independent Monitoring (SIM) team. The use of mobile technology for humanitarian programming in Syria: potential and constraints [Online Report]. UK: UK Foreign, Commonwealth and Development Office; 2017 [cited 2021 26 October]. Available from: <https://www.alnap.org/help-library/the-use-of-mobile-technology-for-humanitarian-programming-in-syria-potential-and-0>.

27. UNHCR. Connecting Refugees: How Internet and Mobile Connectivity can Improve Refugee Well-Being and Transform Humanitarian Action [Online Report]. Geneva: UNHCR; 2016 [cited 2021 27 October]. Available from: <https://www.alnap.org/help-library/connecting-refugees-how-internet-and-mobile-connectivity-can-improve-refugee-well-being>.
