# Supplementary material for: Localisation of digital health tools used by displaced populations in low and middle-income settings: a scoping review and critical analysis of the Participation Revolution
Source: Confl Health. 2023 Apr 15;17:20. doi: 10.1186/s13031-023-00518-9 (PMC10105546; doi:10.1186/s13031-023-00518-9)
Supplement: Supplementary file 4 — Additional file 4. Reflexivity statement. [file 13031_2023_518_MOESM4_ESM.docx]

**Additional Material 4: Research team and reflexivity**

*Authors:*

Jennifer Benson [benson@leibniz-bips.de](mailto:benson@leibniz-bips.de) ^(1, 2, 3)^

Tilman Brand [brand@leibniz-bips.de](mailto:brand@leibniz-bips.de) ^(1)^

Lara Christianson [christianson@leibniz-bips.de](mailto:christianson@leibniz-bips.de) ^(1)^

Meret Lakeberg [lakeberg@leibniz-bips.de](mailto:lakeberg@leibniz-bips.de) ^(1, 3)^

*Organisational affiliations:*

1. Leibniz Institute for Prevention Research and Epidemiology – BIPS, Department Prevention and Evaluation, Bremen, Germany
2. Leibniz Science Campus Digital Public Health, Bremen, Germany
3. University of Bremen, Faculty of Human and Health Sciences, Public Health, Bremen, Germany

| *Characteristics* | *Description* |
| --- | --- |
| Credentials | All authors have academic degrees: JB and LC hold Master of Science degrees, ML holds a Bachelor of Arts degree, and TB holds a doctoral degree. JB is a doctoral candidate. |
| Occupation | All authors are employed at the Leibniz Institute for Prevention Research and Epidemiology (BIPS). JB and TB are researchers. ML is a student assistant and LC is a Librarian. |
| Age, race, gender, socio-economic status & language skills | The research team consisted of three female and 1 male cis-gendered authors, aged between 20 and 49 years (mean age bracket 40-49). All identified as white, and non-disabled with either high or middle socioeconomic statuses. Collectively they hold British, German and US citizenship and speak English, German, French and some Portuguese and Dutch. One team member preferred not to submit certain information here. |
| Relevant professional experiences | Between them, the research team have experience in co-authoring papers on quantitative and qualitative research, articles and reviews in health sciences and social sciences. The majority of these have occurred in Germany and the UK. |
| LMIC experiences | The research team has a broad portfolio of relevant experiences of living and working in LMIC [Tanzania, Ethiopia, South Sudan, Iraq, Syria, Gaza, Lebanon, Pakistan & Bangladesh]. These experiences include professional humanitarian health response activities in emergencies as well as cooperation with LMIC scientific colleagues including personnel exchanges, workshops, and joint publications in the field of public health research. Additionally, the team brings experience in supporting refugees with social services within Europe. |
