# Supplementary material for: Localisation of digital health tools used by displaced populations in low and middle-income settings: a scoping review and critical analysis of the Participation Revolution
Source: Confl Health. 2023 Apr 15;17:20. doi: 10.1186/s13031-023-00518-9 (PMC10105546; doi:10.1186/s13031-023-00518-9)
Supplement: Supplementary file 6 — Additional file 6. The Emerging Indicators of the Participation Revolution from the GMI Dimensions of Localisation Framework [45] and the Participation Key Performance Indicators from the NEAR Localisation Performance Measurement Framework [69]. [file 13031_2023_518_MOESM6_ESM.docx]

**NEAR Localisation Performance Measurement Framework – Participation Revolution Key Performance Indicators (1)**

- Participation of affected people in humanitarian response:
- Affected people are actively involved in assessment of needs, and have a say in how assistance is prioritised, the nature and quality of the assistance and the identification of beneficiaries
- Affected people have information about the implementing agency and have a good knowledge of what the programme is seeking to achieve and who it will benefit
- Affected people are actively asked for feedback during and after the assistance provision and have a means of making suggestions or providing feedback
- Engagement of affected people in humanitarian policy development and standard-setting:
- Deliberations and decisions of humanitarian leadership and coordination forums are informed by in-depth situational understanding, including the views of affected people
- Humanitarian policies and standards are informed by the experience and voices of the affected people

**Emerging Indicators of the Participation Revolution, as mentioned within the Global Mentoring Initiative’s Localisation in Practice Framework (2)**

- Crisis responses are designed, implemented and reviewed in ways that are empowering for affected populations
- People have an early say in the design and planning phase of response
- Formal communication, feedback and response mechanisms are set up with participation from the community and are regularly tested
- Crisis-affected populations are involved in reviews and evaluations
- All people are treated with full human dignity
- Expected standards of staff behaviour are widely known
- The collaborating agencies demonstrate practical competency in working with conflict-sensitivity
- Community/ survivor-led funds are utilised where conditions allow
- Donors and operational agencies plan for adaptation

Reference:

1. NEAR NGO. Localization Performance Measurement Framework (LPMF) 2019 [18 July 2022]. Available from: <https://www.near.ngo/lpmf>.

2. Van Brabant. K., Patel. S. Localisation in Practice: Emerging Indicators & Practical Recommendations [Report]. Washington DC: Global Mentoring Initiative; 2018 [cited 2021 3 August]. Available from: <https://reliefweb.int/sites/reliefweb.int/files/resources/Localisation-In-Practice-Full-Report-v4.pdf>.
